# Controlled human malaria infection in adults identify combinations of merozoite antigens associated with clinical immunity

**DOI:** 10.1038/s41467-026-72716-x

**Published:** 2026-05-05

**Authors:** Rodney Ogwang, Irene N. Nkumama, Kennedy Mwai, Dennis Odera, Lydia Nyamako, Kristin Fürle, Micha Rosenkranz, Rinter Kimathi, Patricia Njuguna, B. Kim Lee Sim, Mainga Hamaluba, Roland Frank, CHMI-SIKA Study Team, Irene N. Nkumama, Kennedy Mwai, Rinter Kimathi, Patricia Njuguna, Mainga Hamaluba, Zaydah de Laurent, Silvia Kariuki, Domitila Kimani, Khadija Said Mohammed, Moses Mosobo, Abdirahman I. Abdi, Donwilliams Omuoyo, Edward Otieno, Jimmy Shangala, Juliana Wambua, Janet Musembi, Jennifer Musyoki, Michelle Muthui, Jedidah Mwacharo, Francis Ndungu, Joyce M. Ngoi, Johnstone Makale, Rodney Ogwang, Omar Ngoto, Dennis O. Odera, Yonas Abebe, Thomas L. Richie, Peter F. Billingsley, Stephen L. Hoffman, Eric R. James, Faith H. A. Osier, Kevin Marsh, John Ong’echa, Peter C. Bull, Sam Kinyanjui, Cheryl Kivisi, Bernhards Ogutu, Fredrick Olewe, Thomas N. Williams, Melissa C. Kapulu, Philip Bejon, James Tuju, Melissa C. Kapulu, Philip Bejon, James Tuju, Faith H. A. Osier

**Affiliations:** 1 Centre for Geographic Medicine Research (Coast), Kenya Medical Research Institute-Wellcome Trust Research Programme, Kilifi, Kenya; 2 College of Health Sciences, Makerere University, Kampala, Uganda; 3 Centre of Infectious Diseases, Heidelberg University Hospital, Heidelberg, Germany; 4 Sanaria Inc., Rockville, MD USA; 5 Modernising Medical Microbiology, University of Oxford, Oxford, UK; 6 Department of Life Sciences, Imperial College London, London, UK; 7 Centre for Tropical Medicine and Global Health, Nuffield Department of Medicine, University Oxford, Oxford, UK; 8 Centre for Clinical Research, Kenya Medical Research Institute, Kisumu, Kenya; 9 Department of Pathology, University of Cambridge, Cambridge, UK; 10 Pwani University, P. O. Box 195-80108 Kilifi, Kenya; 11 Center for Research in Therapeutic Sciences, Strathmore University, Nairobi, Kenya; 12 Department of Medicine, Imperial College London, London, UK

**Keywords:** Recombinant vaccine, Humoral immunity

## Abstract

Two vaccines are currently licensed against *Plasmodium falciparum* malaria but offer only partial protection that wanes, necessitating repeated dosing that is challenging to implement where it is needed most. We previously identified a subset of Kenyan adults with naturally acquired clinical immunity using controlled human malaria infection studies (CHMI, *n* = 86/142; ClinicalTrials.gov - NCT02739763). Subsequent studies showed that clinical immunity was strongly correlated with IgG Fc-mediated functional activity against whole merozoites. Here, to zoom in on specific targets associated with clinical immunity, we use samples from the CHMI study and a custom protein microarray (KILchip) to measure IgG antibody reactivity against seventy recombinant merozoite antigens. We identify the top-ranking antigens by analyzing the data using five distinct statistical methodologies: the nonparametric Wilcoxon rank sum test, a Cox proportional hazards model, a modified Poisson regression model, and the machine learning Least Absolute Shrinkage and Selection Operator (LASSO) and random forest models. Antibodies against MSP1, MSP11, RAMA, MSP7, PF3D7_1401600 (PHISTB) and PTEX150 were consistently associated with clinical immunity and in combination predicted complete protection. These findings may be useful to prioritize the next generation of malaria blood stage vaccine candidates.

## Abstract (web summary)

This study used a controlled human malaria challenge study in African adults and a custom merozoite protein microarray to identify antigens associated with clinical immunity against *P. falciparum* malaria for further study and development.

## Introduction

Malaria remains an urgent public health problem with ~249 million clinical cases globally per year^1^. The highest burden is in the WHO Afro region (94%), and children under the age of five years account for three of every four cases^1^. Although a wide range of tools have been applied to control malaria, the clinical burden has remained essentially unchanged for the last decade. This stagnation of progress has been attributed in part to the rise of pyrethroid resistance in insecticide treated nets (ITNs), drug resistant parasites, as well as health and humanitarian emergencies such as famine, flooding and disease outbreaks^2^.

The first malaria vaccine was licensed in October 2021 (Mosquirix), and a second (R21) followed two years later. Both vaccines target the sporozoite stage of the parasite that is transmitted between mosquitoes and humans. Both vaccines are partially protective and have variable efficacy that wanes over time^3–5^. The WHO preferred Target Product Profile (TPP) for second generation vaccines recommends >90% efficacy with a duration of protection of at least one year^6^.

People living in areas with a high intensity of malaria transmission gradually acquire immunity following repeated malaria infections. This naturally acquired immunity (NAI) is characterized by an ability to control parasitaemia during the blood stage of the infection, and to prevent the clinical symptoms of malaria^7,8^. Passive transfer studies in humans provided the strongest evidence that antibodies targeting blood stage parasites were key mediators of NAI. Gamma globulin (IgG) from adults who had acquired immunity was successfully used to treat patients hospitalized with malaria^9–12^. In contemporary CHMI studies, we and others have demonstrated that NAI controls parasite growth and prevents the development of clinical symptoms^13–15^.

We previously found that IgG Fc-mediated antibody function in conjunction with complement, neutrophils, natural killer cells and monocytes was superior to the growth inhibition assay in predicting protection against parasite growth and clinical symptoms of malaria in challenge studies of adults with varying degrees of naturally acquired immunity^16–18^. We showed that antibodies against full-length MSP1, the most dominant antigen on the surface of the merozoite, recapitulated approximately 80% of the protection that we observed in studies using whole merozoites^19^. Multiple previous studies suggested that the breadth of antibody response, i.e. the number of distinct antigens recognized is strongly associated with protection^20,21^. We therefore sought to identify merozoite antigens, that may independently and or in combination be associated with greater protection to clinical malaria, with the aim of informing further pre-clinical and clinical development.

Here we used KILchip, a custom protein microarray designed to facilitate the prioritization of merozoite stage antigens for vaccine development^22^ to probe samples from the CHMI study, asking whether responses to individual proteins were associated with protection. We applied five distinct analytical approaches to the multidimensional immunological data to identify responses that were consistently most strongly associated with protection across methodologies^22^.

## Results

### CHMI enables the accurate capture of study exposures and endpoints

**Fig. 1:**
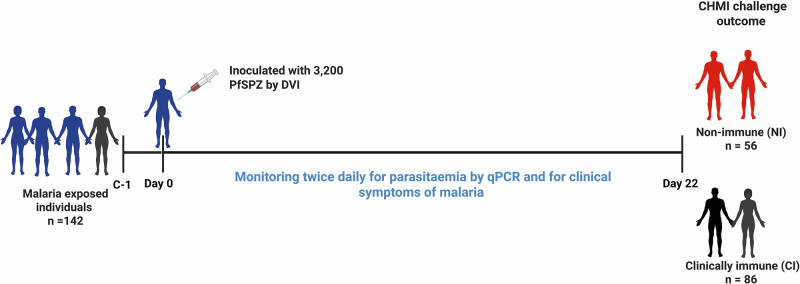
Study design. Kenyan adult volunteers (*N* = 142) were infected with 3,200 live *P. falciparum* sporozoites via direct venous injection (DVI). Parasitaemia was quantified by qPCR from day 7 to 21. Volunteers were treated if parasitaemia exceeded 500/μl of blood, or if they developed a fever (axillary temperature ≥37.5 °C) with parasitaemia of any density. A proportion were subsequently classified as non-immune (NI, *n* = 56, red) as they required treatment following challenge. The remainder were classified as clinically immune (CI, *n* = 86, black) and did not meet the treatment criteria over the 22 days of follow up. qPCR: quantitative Polymerase Chain Reaction^13,17^. The figure Created in BioRender. Ogwang, R. (2026) https://BioRender.com/0i0sr5y.

One hundred and forty-two adults were challenged with ~ 3200 sporozoites and monitored twice daily for the development of clinical symptoms and for parasitemia over 22 days. Treatment with Artemether-Lumefantrine was provided if any volunteer developed clinically important symptoms, if parasitaemia exceeded 500 parasites/µl, or at the end of the study. Fifty-six of one hundred and forty-two volunteers (39%) met the treatment criteria before the end of the study and are hereafter referred to as non-immune (NI, Fig. 1 and S1). The remaining eighty-six volunteers that did not meet these criteria are hereafter referred to as clinically immune (CI, Fig. 1).

### IgG antibody response patterns were heterogenous

**Fig. 2:**
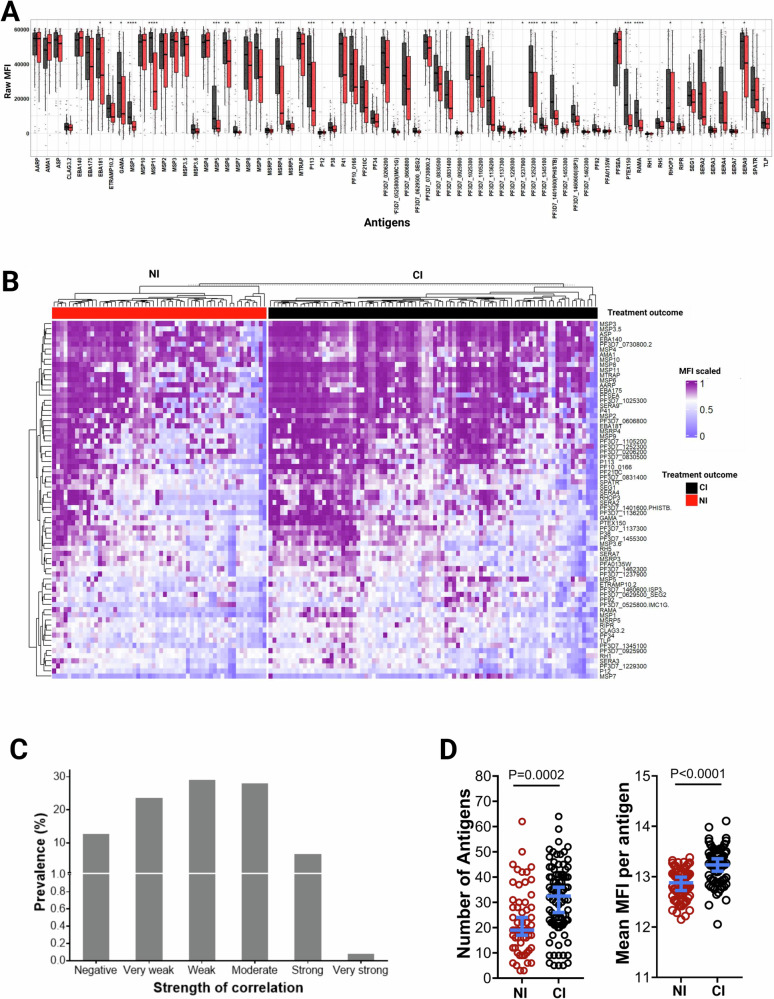
Antibody responses are heterogenous and not strongly correlated. **A** Box plots showing median interquartile of range of relative antibody levels (MFI) before normalization for the different antigens (*n* = 70) investigated categorized by clinically immune (CI, *n* = 86, black), and non-immune (NI, *n* = 56 red). Mann Whittney U testing with significance shown as *, *p* < 0.05; **, *p* < 0.01; ***, *p* < 0.001. **B** Unsupervised clustering heatmap of microarray data. Treatment outcomes are annotated in the top two rows: clinically immune (CI, black), non-immune (NI, red). Each column represents one volunteer while each row represents a single antigen. The mean fluorescence intensity (MFI) for each of the protein tags (CD4, GST and MBP) was calculated and subtracted from the respective antigens. Data were batch corrected, normalized by variance stabilizing normalization (VSN)^43^ and scaled before analysis. **C** Pairwise correlations between responses assessed using the non-parametric Spearmans r correlation matrix. R values were classified as follows: negative (<0), very weak (0.0-0.19), weak (0.20-0.39), moderate (0.40-0.59), strong (0.60-0.79) and very strong (≥0.80). **D** The number of antigens recognised by plasma from each volunteer (geometric mean (95%CI)) (left) and mean MFI values (median (IQR)) per antigen for the 70 antigens analyzed (right) was compared between volunteers who were NI (*n* = 56 red) versus CI (*n* = 86 black),. Error bars represent the median and 95% confidence interval. p values were calculated using the Mann-Whitney U test, p ≤ 0.05 considered significant.

Antibody data are reported for 70/95 KILchip antigens that satisfied the quality control criteria reported under statistical methods. The list of antigens thus analyzed is provided in (Table S1). The level of antibody reactivity against each antigen varied; some antigens showed a high level of reactivity and were present in a high proportion of volunteers, while responses to others were less strong and detected in fewer volunteers (Fig. 2A). We previously explored four functions for normalization (variance stabilization normalization (VSN), Log_2_, robust-linear-model (RLM) and cyclic loess) to identify one that optimally reduced the mean–variance dependence (MVD)^23^. The cyclic loess and RLM were not optimal. Consequently, we adopted the VSN normalization approach that overcomes the limitations of log transformations minimizing the inflated variance around low signal intensities, but also results in normalization as effectively as log-transformation^23^. Hereafter, all analyses were conducted using VSN normalized data (Fig. S2). An unsupervised clustering analysis of the microarray data revealed variation in the antibody response patterns between volunteers and between antigens with volunteers displaying diverse combinations of antibodies (Figs. 2B and S2). Antibody response patterns from CI and NI volunteers clustered to the left and right of the heatmap, respectively. A Spearman analysis for collinearity showed that the correlation between responses to most antigens was either nil or very weak to modest (r = 0 to 0.6, Fig. 2C). Strong correlations (r > 0.6) were observed for <10% of all antigen pairs. The breadth (number of individual high responses) and magnitude (sum of mean fluorescence intensity, MFI, calculated across all antigens) of antibody responses was correlated with the clinical outcome. Volunteers that were subsequently classified as NI had high responses to a median of 19 antigens (range 3-62) compared to 33 antigens (range 5-64) for the CI (Fig. 2D, left panel). Likewise, the magnitude of the total reactivity across all antigens was lower in the NI versus CI groups, p < 0.0001 (Fig. 2D, right panel).

### Independent analytical approaches identify six antigens consistently associated with protection

We used five analytical approaches to identify the top responses associated with protection. Protection was defined as remaining clinically well and controlling parasitaemia below the predefined threshold of 500 parasites/µl following challenge (as described above).

**Fig. 3:**
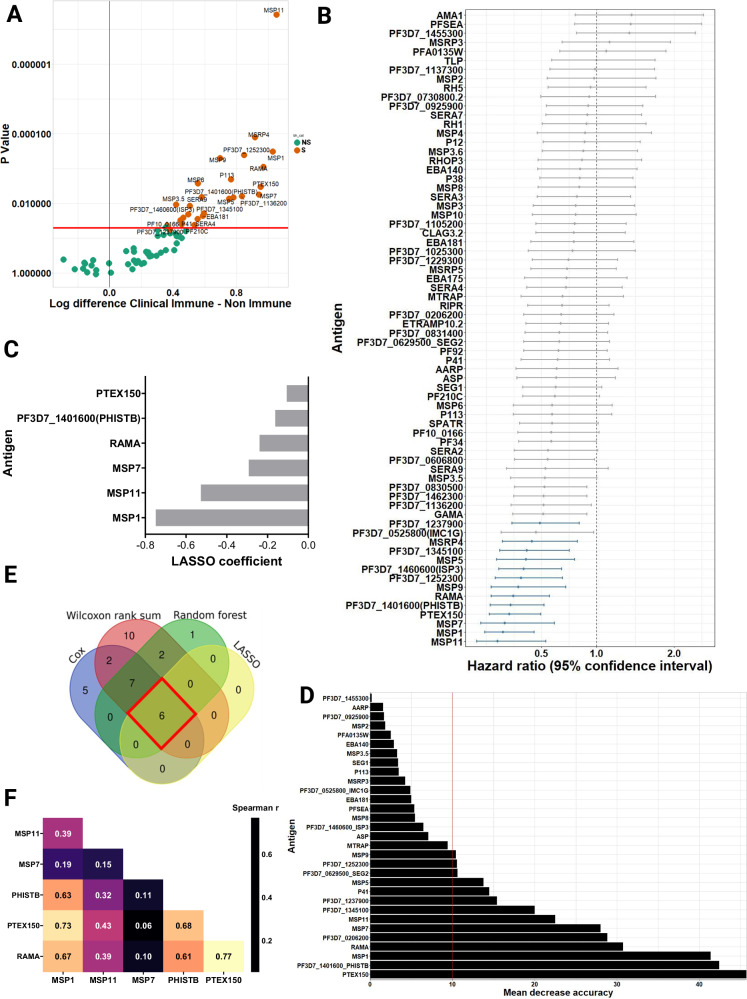
Independent analyses identify six antigens consistently associated with protection. **A** Wilcoxon rank-sum test P values adjusting for multiple comparisons using the Benjamini-Hochberg (BH) correction for normalized mean fluorescence intensity (MFI) values obtained from the KILchip microarray^22^. The volcano plot shows the log mean difference between clinically immune (CI, *n* = 86 black), and non-immune (NI, *n* = 56 red) volunteers on the x axis and the adjusted p values on the y axis. The red horizontal line shows the cut-off for significance (p < 0.05). Each dot represents a specific antigen; green P > 0.05 and orange p < 0.05. **B** Cox regression model adjusting for potential confounders (antimalarial drugs levels in plasma, dantu genotype, α-thalassemia genotype and year of study). The black dotted line represents a hazard ratio (HR) of 1. The dots represent the point estimate for antibody responses against each antigen. The error bars lines illustrate the 95% confidence intervals. Error bars in blue represents p values that remained significant (p ≤ 0.01) after BH correction for multiple comparisons. **C** Least absolute shrinkage and selection operator (LASSO) analysis. The LASSO coefficients are shown on the x axis for the 6 antigens prioritized. **D** Random forest analysis optimized to identify important antigens that differentiate between clinically immune (CI, *n* = 86 black), and non-immune (NI, *n* = 56 red) volunteers. The mean decrease in accuracy for selected antigens is shown. Antigens with a mean decrease accuracy greater than 10% were identified as important for protection. **E** Venn diagram consolidates the 33 antigens selected by the different analytical approaches (**A**–**D**) above. **F** Heat map showing the non-parametric Spearman correlation matrix for the top six antigens.

We started with a univariable nonparametric Wilcoxon rank-sum test adjusted for multiple comparisons using the Benjamini-Hochberg (BH) correction. Antibodies to 27 *P. falciparum* antigens were significantly higher in CI compared to NI volunteers (P value < 0.05, Fig. 3A and Table S2). Notable antigens identified in this first analysis included well-studied merozoite surface proteins 1 (MSP1), but not apical membrane antigen 1 (AMA1), MSP-3 or PfRh5. Several novel antigens identified in our antigen discovery program^22^ but not yet studied in the context of immunity such as Pf3D7_1252300 and Pf3D7_1345100 were also significantly higher in CI compared to NI volunteers, p < 0.0001 and *p* = 0.0033, respectively.

Second, we used a proportional hazards model (Cox regression) to ask whether having high levels of antibodies to individual antigens was associated with a longer time to the clinical episode of malaria compared to having low levels. Each analysis was adjusted for potential confounders including lumefantrine drug levels, dantu genotype, year, and α-thalassemia genotype^24^. We found that high levels of antibodies to 20 antigens were associated with a longer time to the clinical episode of malaria than lower levels, and these are displayed in ascending rank order in (Fig. 3B). The well-studied MSP-1 antigen was among the best performing antigens in this analysis, in addition to antigens that are less well-studied in the context of acquired immunity such as MSP-11 and MSP7, and some novel antigens^22^ such as Pf3D7_1252300 and Pf3D7_1460600 (ISP3). As previously observed in the Wilcoxon rank sum analysis (above), antibodies to well-studied antigens such as AMA1 and PfRh5 were not significantly associated with the time to malaria episode.

In the third analytical approach we used a Poisson regression model to test whether having high levels of antibodies to individual antigens was associated with a lower risk of developing malaria (i.e. being CI). Each model was adjusted for the same confounders in the Cox regression analysis above. The results we obtained were very similar to those observed for the Cox regression analysis, with minor variations in the rank order of a few antigens. In addition, antibodies against a handful of antigens were identified as important in the Poisson but not the Cox regression, and vice-versa, but these tended to rank among the lowest of significant associations (Fig. S4).

The fourth analytical method was the machine learning least absolute shrinkage and selection operator (LASSO). This penalized regression method minimizes prediction error while keeping the model simple in terms of number of predictors between the two outcome groups, NI and CI. We first selected the optimal lambda through a cross-validation analysis that involved sampling with replacement of 15 variables at a time (Fig. S5). We then applied this model to identify the minimum number of antigens that best discriminated NI from CI volunteers. Six antigens were thus identified and included MSP1, MSP11, RAMA, MSP7, PF3D7_1401600 (PHISTB) and PTEX150, in order of importance (Fig. 3C).

The fifth and final method was the machine learning random forest analysis that prioritizes antigens based on their ability to discriminate NI versus CI volunteers. A random forest model was fitted to the data with bagging, i.e. repeatedly sampling from the dataset with replacement, fitting decision trees for each out-of-bag sample, and noting the out-of-bag error^25^. The model was then optimized using a 10-fold cross validation loop to get the lowest out-of-bag error and highest accuracy. The best model had 10,000 trees with 48 random antigens per tree. The average error for the forest was analysed and antigens prioritized by comparing the out-of-bag error before and after permutation over all trees and assigning a score. The score was normalized over the standard deviation and the antigens with the highest scores were ranked as the most important. Sixteen antigens with a mean decrease in accuracy above an arbitrary 10% cut-off were thus prioritized as important in differentiating between NI and CI volunteers. Notably, all the antigens identified in the LASSO analysis, were independently selected in the random forest, in addition to a further ten antigens (Fig. 3D).

Overall, a total of 33 antigens were identified as associated with protection across five analytical methods (Table S2). Of these, six were consistently ranked at the top in all methods; MSP1, MSP11, RAMA, MSP7, PF3D7_1401600 (PHISTB) and PTEX150 (Fig. 3E, and Table S2). The pairwise correlation coefficients between responses to PTEX150, MSP1, PHISTB and RAMA were strong (r = 0.61 - 0.77), while those against MSP7 and MSP11 were weak (Fig. 3F).

### Combination analyses predict complete protection

**Fig. 4:**
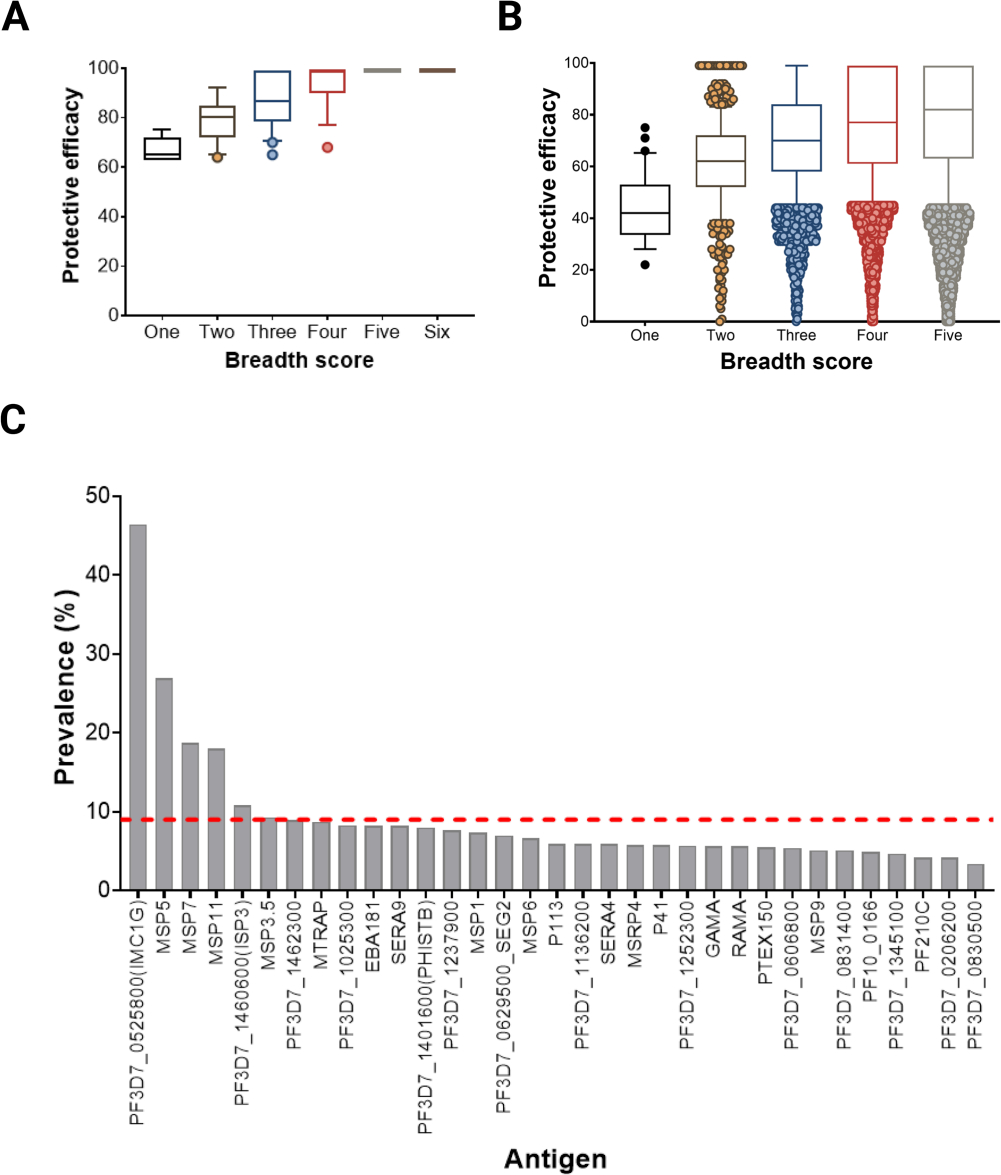
Combination analyses predict complete protection. **A** Combination analysis using only the six antigens that were consistently selected as important for protection. The relative risk of being CI (*n* = 86) versus NI (*n* = 56) was compared between individuals with high versus low-to-moderate antibodies for each combination using a modified Poisson regression model. The protective efficacy (1-RR) x 100) % for each combination was then calculated and the median (IQR) plotted. **B** Combination analysis in the top 33 antigens identified as important across all analytical methodologies. **C** The frequency of occurrence for each of the 33 selected antigens in 913 combinations of two or three antigens that were associated with 100% protective efficacy. The red dotted line shows a frequency of occurrence of 9% that would be expected if there was equal chance of occurrence for each antigen.

We and others have previously shown that combinations of responses were better predictors of protection than single antigens^20^. We therefore tested all possible combinations of responses to 2, 3, 4 and 5 antigens. We first tested combinations of the six antigens consistently ranked at the top (Fig. 3E) and thereafter explored the 33 antigens identified as important across all analytical methodologies. For both analyses, protective efficacy (defined as (1 – RR) %) increased with breadth, defined as the sum of individual antibody responses. In the first analysis with only the top six antigens, responses to a combination of four antigens was associated with a median protective efficacy of 100% (Fig. 4A). In the second analysis including 33 antigens, rare combinations of responses to just two or three antigens were associated with complete protection (Fig. 4B).

We asked which antibody responses were most effective in the combinatorial analyses by examining which antigens were most frequently involved in combinations that provided 100% efficacy (Fig. S6). Nine hundred and thirteen combinations of 2 or 3 responses were associated with 100% protective efficacy. The frequency of any antigen being randomly included in any of these protective combinations is 9%. We found that responses to PF3D7_0525800(IMC1G) occurred in almost half (46%) of 913 combinations (Fig. 4C). Four other antigens were also present more frequently than by chance: MSP5 (27%), MSP7 (19%), MSP11 (18%) and ISP3 (11%).

### Prioritized antigens are highly conserved and likely essential

Highly conserved and essential genes are attractive as vaccine candidates. We analyzed genetic variation data on PlasmoDB (release 68, 07/05/2024). We compared our prioritized shortlist of antigens against PfRh5, a leading blood stage vaccine candidate considered to be highly conserved^26–28^. MSP1 is highly polymorphic and under high immune pressure with a non-synonymous to synonymous single nucleotide polymorphisms ration (dN/dS) of 3.59 compared to 0.73 for PfRh5. The remaining antigens prioritized in our analysis (5/6) of those consistently identified across all methodologies and 5/5 of those occurring at a frequency of >10% in combinations had dN/dS ratios between 0.25 (ISP3) and 2.9 (MSP7). Further their non-synonymous SNPs per coding sequence length ratios were MSP11 (0.0074), MSP7 (0.027), MSP5 (0.0073), IMC1G (0.005), and ISP3 (0.0022) compared to 0.01 for PfRh5 suggestive that they are relatively conserved (Table S3).

Data on essential *P. falciparum* genes was available from previous studies using piggyBac insertion saturation mutagenesis^29^. We found that for the top 33 antigens selected the mutagenesis index score (MIS) was <0.5 for most antigens which suggests that they are likely essential (Table S3). Likewise, the mutant fitness score (MFS) was <-2, indicating a growth fitness cost for mutating the genes. For the top six antigens identified using multiple analytical strategies (MSP1, MSP11, RAMA, MSP7, PF3D7_1401600 (PHISTB) and PTEX150,) as well the five antigens identified through our combinational analyses (MSP11, MSP7 MSP5, IMC1G and ISP3) the MIS ranged from a low of 0.14 for MSP1 to 1.0 for RAMA, PTEX150, while MFS ranged from -3.19 – MSP1 to -1.47 -RAMA (Table S3). Both the mutagenesis index and the mutant fitness scores were comparable to PfRh5 (MIS = 0.126 and MFS = -3.14)^29^.

## Discussion

Our study leverages three distinct methodological advances to improve our understanding of the merozoite proteins that drive naturally acquired immunity against malaria. First, unlike the traditional prospective longitudinal cohort studies that we and others have used previously, we used human challenge studies that have clear advantages for the type of analysis we conducted. In the latter, both the exposure (i.e. challenge dose, strain and timing) and the outcome (clinical episodes of malaria) variables are accurately controlled in the study design, minimizing misclassification bias at multiple levels and enhancing the precision of the estimates of protection^24^. Second, previous studies have either examined a small number of antigens, or have undertaken systematic screens of >1000 antigens applying a hypothesis-independent approach including as much of the *P. falciparum genome* as possible^30–34^. We favored a hypothesis-driven approach reasoning that proteins either directly localized on the surface of the merozoite, or secreted onto its surface at the time of erythrocyte invasion, are accessible to antibodies and therefore likely targets of protective immunity^22^. Over 95% of the antigens on our KILchip microarray are immunogenic compared to ~10% of the antigens included on other platforms^22,30,31,33–35^. Third, individual studies typically employ a single analytical method, which may be re-applied to data from a different population or experiment for validation. A review of the relevant literature reveals a multiplicity of analytical approaches, making it challenging to compare results across studies, and furthermore difficult to know if discoveries are contingent on statistical methodology. We therefore analyzed our data using multiple statistical approaches reported in literature, comprising three widely used traditional methods and two newer machine-learning based algorithms.

Six merozoite antigens were consistently identified as strongly associated with protection using the five analytical methods: MSP1, MSP11, MSP7, RAMA, PTEX150 and PF3D7_1401600 (PHISTb). The pairwise correlation coefficients between responses to PTEX150, MSP1, PHISTB and RAMA were strong (r = 0.61 - 0.77), suggesting that not all these four may be independent predictors of outcome, while correlation coefficients including MSP7 or MSP11 were weak, increasing the likelihood that these are independent predictors. We complemented this output with combinatorial analyses including antibodies against all antigens that were significantly associated with protection in the individual analytical approaches (*n* = 33). We found that none of the individuals with high responses to selected pairs or trios of antigens (913 combinations) from this larger pool succumbed to malaria (100% protection). We do not suggest that these combinations would likely translate to 100% protection in the field, the finding of complete protection in our setting relates to a controlled inoculum in 142 adults, and hence refers to our observed outcome which justifies the prioritization of these antigens. Vaccine efficacy would need to be measured in a trial.

Responses to PF3D7_0525800 (IMC1G), occurred in almost half (46%) of all combinations. Antibodies against MSP5 (27%), MSP7 (19%), MSP11 (18%) and ISP3 (11%) were detected more frequently than by chance. Interestingly, only antibodies against MSP7 and MSP11 were prioritized in both the multi-method and combinatorial analyses. An analysis of dN/dS ratios against those of the highly conserved PfRh5 antigen (0.73) revealed that except for MSP1 (3.59) and MSP7 (2.9.), the remaining antigens had lower ratios (<7.33) indicating they could be classified as highly conserved, an important consideration for vaccine design. With the exception of PTEX, RAMA and PHISTB, most of the prioritized antigens can be categorised as likely essential based on the mutagenesis index and mutant fitness scores^26^.

Of the antigens prioritised, MSP1 has been extensively studied as a vaccine candidate and largely abandoned due to the lack of protection observed in early phase clinical trials^36^. However, these studies used a fragment (i.e. MSP1-19) accounting for approximately 10% of the full-length protein in contrast to our study which used a correctly folded version of full-length MSP1^22^. It is one of the most abundant antigens on the surface of the merozoite^37^ and is critical to parasite invasion of the red cell. Recent studies indicate that full-length MSP1 induces IgG Fc-mediated function via multiple immune effectors in both naturally acquired and vaccine-induced immunity^19^. Functional studies using samples from the CHMI study revealed that antibodies against merozoites and recombinant full-length MSP1 were highly correlated and induced Fc-mediated function that was significantly associated with protection from malaria^17,19^. MSP7, MSP11, MSP5 and RAMA are likewise thought to be involved in parasite invasion of the red cell, and previous cohort studies have suggested correlations with protection in children. PTEX150 and PfHISTB are less well studied antigens in the context of immunity. PfHISTB does not appear to have a function in invasion, but rather is part of the *Plasmodium* helical interspersed sub telomeric (PHIST) b protein family and has a function in mediating the rigidity of the infected red cell membrane^38,39^. PTEX150 is part of the *Plasmodium* translocon of exported proteins (PTEX) complex on the parasitophorous vacuole membrane and plays a crucial role in protein export to the infected red blood cell membrane^40,41^. More recently it has been shown to also be essential for parasite growth in hepatocytes^42^. At the merozoite stage, PTEX150 is localized within dense granules in merozoites alongside other members of the PTEX complex which are later released into the parasitophorous vacuole^43^. However, the secretion or localization of PTEX150 on the merozoite surface during the invasion process is unknown. IMC1g and ISP3 have only been recently reported as part of an antigen discovery program^22^ and warrant further study.

A vaccine strategy combining 6 antigens is likely not tractable given the challenges of multi-antigen formulations. However, our combinatorial analyses indicated that two or three antigens may be sufficient for a high level of protection. Two of the six antigens identified as important in the five independent analytical approaches were identified more frequently than by chance in important combinations: MSP7 and MSP11. Responses against these two antigens were also the least correlated. The impact of combinations will depend on the prevalence of responses and the collinearity with other independent predictors of outcome as well as the magnitude of associations. Responses to RAMA and PTEX150 were not well represented in the combination analysis, perhaps reflecting their strong collinearity with MSP1. Additional data on antibody function for the remaining antigens would strengthen the evidence for their prioritization for vaccine development.

Given the complexities of vaccine development, the antigens that we have identified as having potential for combination or multivalent vaccines will need further reduction and refinement. We consider these data as important first steps. Further epitope mapping and structural level characterization will need to be applied to elucidate and subsequently validate the critical epitopes necessary for protection and amenable to vaccine development. Further studies would include vaccination of mice to determine functional immunity and epitope and structural level characterization of these antigens may elucidate the critical epitopes required to mediate protection. Antigens raising non-functional responses would be de-prioritized, and further challenge studies with multiple combinations would identify the minimum number of antigens required for further clinical development. We would prioritize testing combinations of 2 or 3 antigens that were associated with the highest levels of protection in our study.

Limitations of the study include the retrospective finding of low levels of lumefantrine in some of the participants^24^. As previously done, we excluded those with levels of lumefantrine above the minimum inhibitory concentration from the main analysis. Previous findings indicated that levels of lumefantrine below the minimum inhibitory concentration do not alter parasite growth in vivo, as would be expected given the in vitro definition of a minimum inhibitory concentration. However, we also undertook a sensitivity analysis (Fig. S7), where we excluded all volunteers with any detectable levels of lumefantrine, and this analysis confirmed that the top antigens remained highly associated with protection. Another limitation is although we identify combinations of antigens associated with protection we do not show whether these specific responses may increase the potency of the IgG Fc-mediated response that we have previously shown important for protection. Indeed, future studies using purified antigen specific IgGs, monoclonal antibodies, or serum depletion experiments are required to investigate the functional potency of these identified antigens. The microarray was designed to investigate merozoite antigens, however as the challenge was IV PfSPZ similar studies investigating sporozoite specific antigens may improve our understanding.

Our approach has arguably maximized specificity over sensitivity: i.e. we have more confidence “ruling in” antigens than “ruling out”. In focusing on the six antigens that were consistently identified, it is likely that we are excluding antigens that were genuinely associated with outcome but where methodological issues led to erroneous exclusion. For example, one possibility of insensitivity to markers of protection is saturation of signal. This is suggested for the following antigens (AMA1, EBA140, EBA175, EBA180, MSP2, MSP3, and MSP4), where distributions were truncated at the upper limits of the assays dynamic range. In addition, our analyses may suffer from over-fitting, misclassification biases or over-simplification. Especially as regards the combination analysis where multiple antigens are possible. We do not suggest that any combination are likely to be associated with 100% protection, rather we are using this analysis to highlight combinations of antigens that merit further investigation. Furthermore, responses to some antigens may be inaccessible to study through naturally acquired immunity if the prevalence or magnitude of responses raised by natural exposure are too low. Hence, we would focus interest on selecting these antigens for further development as vaccine candidates rather than excluding other antigens, particularly where there are other lines of evidence (for instance the antigen RH5 is essential for red cell invasion, low levels of antibodies are identified from naturally acquired immunity, and there is evidence that RH5 antibodies conferred by vaccination are protective). Further studies are underway to assess the functional potency of antibodies against all prioritized antigens, as we have previously done for MSP1^19^.

## Methods

### CHMI-SIKA study

All study volunteers were challenged under the same conditions, strain, dose, and procedures outlined in the study protocol^13^. The study was open, non-blinded, and non-randomized. The participants, all Kenyan and of similar ethnicity, were carefully screened and recruited from different areas of the country to provide varied previous malaria exposure. Those with sickle cell trait, known to influence malaria infections, and active asexual parasites were excluded. The study was conducted in three phases between 2016 and 2018, with 142 participants infected with 3,200 NF54 sporozoites (Sanaria®) through direct venous injection (DVI) (Table S4). Blood stage parasitemia was monitored by quantitative polymerase chain reaction (qPCR) twice a day from day 7 to day 14, and then once daily from day 15 to day 21 post-challenge. The study endpoint was met when parasitaemia exceeded 500 parasites/µl of blood, or if signs and symptoms of malaria developed with any level of parasitaemia, or at 21 days post-challenge when the study ended^13,14^. Our primary outcome was the need for treatment before day 21. Volunteers meeting this criterion are referred to as non-immune (NI), while those that did not are considered clinically immune (CI). We analysed our immune responses in relation to parasite density as a secondary outcome. The average parasite density was calculated using the geometric mean between days 8.5 and 22 post-challenge, excluding any time points after treatment. The maximum parasite density refers to the highest value observed during the same period, also excluding any time points after treatment. All immunological tests reported here were performed on plasma samples collected the day before challenge (C-1).

The CHMI-SIKA study was conducted at the Kenya Medical Research Institute (KEMRI)-Wellcome Trust Research Programme in Kilifi, Kenya, with ethical approval from the KEMRI Scientific and Ethics Review Unit (KEMRI//SERU/CGMR-C/029/3190) and by the University of Oxford Tropical Research Ethics Committee (OxTREC 2-16). All participants gave written informed consent. The study was registered on ClinicalTrials.gov (NCT02739763), conducted based on good clinical practice (GCP), and under the principles of the Declaration of Helsinki.

### Protein expression and microarray slide preparation

All proteins were expressed and purified as previously described using either a mammalian or bacterial expression system^22^. For mammalian expression, all sequences were codon-optimized and all potential *N*-linked glycosylation sites (NXS/T) modified by substituting serine or threonine residues with an alanine residue. Plasmids containing codon-optimized genes of interest [Addgene (https://www.addgene.org) or newly synthesized by GeneartAG)] were sub-cloned into a derivative of the pTT3 expression vector (Add gene (https://www.addgene.org) that contained an N-terminal signal peptide derived from the mouse *kappa* light chain to drive secretion of antigen, a rat Cd4 domains 3 and 4 tag and a hexa-histidine tag for protein purification. Proteins were subsequently expressed using the Expi293 expression system (Invitrogen) according to the manufacturer’s instructions. Briefly, Expi293F cells were cultured to a density of 2.0 × 10^6^ cells/ml and transfected with expression vectors using the Expifectamine 293 transfection reagent (Invitrogen). Cells were then incubated at 37°C with 8% CO_2_ in an orbital shaker at 125 rpm. Culture supernatants were harvested 6 days post-transfection, and proteins were purified using Ni-NTA purification columns (Invitrogen).

A minority of proteins were expressed in *E. coli* using pGEX-2T and pMAL-c2X vectors to produce fusion proteins with the carriers glutathione-S-transferase (GST) and maltose-binding protein (MBP), respectively. These were transformed into BL21 (DE3) pLysS *E.coli* cells and expressed as previously described^44–46^. Cell expansion, induction of protein expression and subsequent purification were performed as previously described. Finally, for *Pf* SEA1, the encoding gene was amplified from *P. falciparum* 3D7 cDNA using previously described primers^22^. The PCR products were cloned into pEXP5-NT/TOPO expression vector and transformed into BL21 (DE3)^44–46^ and expressed as above. All purified recombinant proteins were dialysed into phosphate buffered saline and quantified using NanoDrop (Thermo Scientific) before printing onto nitrocellulose slides (ONCYTE SuperNOVA, GraceBio). Proteins were printed in duplicate at a concentration of 250 μg/ml using the Ultra Marathon micro-arrayer (ArrayJet) with the Inkjet printing technology and the command centre 1.5.0.1 (ArrayJet). Printing was carried out at 50% relative humidity and at 18°C followed by drying overnight at 18°C in the arrayer after printing before storage in slide boxes with desiccant at 4°C until use. Print verification was conducted by scanning at a high photomultiplier (PMT) to evaluate printing quality (at the 532 nm wavelength (green channel)) and to visualization landmark spots (at the 635 nm wavelength (red channel)).

### Antibody detection assay

Printed slides were carefully assembled onto the hybridization cassette and sealed using silicone gaskets (ARYC) to form leak-proof individual wells. We modified a published protocol for the detection of antibodies^47^. Briefly, wells were washed thrice with 0.1% Tween 20/HEPES buffered saline (1.4 M NaCl, 50 mM KCl, 20 mM CaCl_2_, 10 mM MgCl_2_, 100 mM HEPES; HBS) followed by HBS to remove any unbound proteins. Non-specific binding to the slide surface was prevented by blocking with 200 μl of 2% BSA/0.1% Tween 20/HBS for 2 h at room temperature while rotating on a microarray hybridization station (ARYC) at 350 rpm. Wells were washed thrice and incubated overnight at 4°C with 150 μl of serum diluted 1:400 and rotating at 350 rpm on the hybridization station. Thereafter, wells were washed as described above and incubated with 150 μl of donkey anti-human IgG-Fcγ fragment specific Alexafluor^647^ for 3 h at room temperature, followed by three washes. Slides were carefully disassembled from the hybridization cassettes, rinsed thrice in distilled water and dried by centrifugation at 300 g for 5 min using a combiSlide adapter (Eppendorf) and stored in slide boxes in the dark. Slides were scanned using a Genepix 4,000 B scanner coupled to the GenePix Pro & Microarray Acquisition and Analysis Software (Molecular Devices).

### Statistical analysis

Statistical analyses were conducted using R version 3.6.1 and GraphPad prism version 8.4.0. The mean fluorescence intensity (MFI) from each spot was cleaned by first subtracting the background fluorescence from each spot. Next, MFI values for each of the protein tags (CD4tag, GST and MBP) were calculated and subtracted from the respective antigens. GST and MBP tags had high MFI values such that post tag subtraction individual responses were negative, preventing accurate interpretation. This may have been due to contaminating bacterial proteins since MBP and GST tagged antigens and the tags themselves were expressed in *E. coli*. Thus, data from 23 antigens tagged with GST and 2 with MBP were excluded from the analysis. The signal level from duplicate spots were compared and where coefficient of variation was greater than 20% the samples were assayed again.

The data from the remaining 70 *P. falciparum* antigens, was batch corrected because the samples were run in different batches and then normalised using variance stabilising normalisation (VSN) before analysis^23^. We previously explored four (VSN, Log2, RLM and cyclic loess) functions for normalization to identify one that optimally reduced the mean–variance dependence (MVD)^23^. We formally assessed this using mean versus standard deviation plots (*meanSdPlots*) and coupled this to automatically derived Spearman correlation estimates (*Rho*) and Cox–Stuart or the Mann–Kendall trend tests. The latter quantifies the performance of the normalization. Implementation of the log_2_ approach led to an inflation of variance for the low MFI values. The cyclic loess and RLM were not optimal. Consequently, we adopted the VSN normalization approach. The VSN method overcomes the limitations of log transformations minimizing the inflated variance around low signal intensities but also results in normalization as effectively as log-transformation. It calibrates between-feature variation through shifting and scaling mechanism in which all the data are adjusted. Huber et al. and Durbin et al. independently proposed the VSN approach for such data which is a variant of the log-transform (*glog2*).

Spearman’s rank correlation was used to examine correlations between variables. Antigen selection was done using 5 analysis methods: 1) The nonparametric Wilcoxon rank sum test with Benjamini-Hochberg correction^48^ for multiple comparisons was performed to compare antigen-specific antibody levels between treated and non-treated volunteers. 2) Cox proportional hazards model adjusting for potential confounders (year of study, dantu and α-thalassemia genotypes, and anti-malarial drug levels) was used to identify antigens significantly associated with a prolonged time to treatment. 3) A modified Poisson regression model^49^ which has a robust error variance and provides relative risk as the parameter of primary interest and assist in convergence problems that sometimes arise with binomial regression models was used to determine the relative risk of treatment (due to clinical symptoms and or parasite densities >500 parasites/µl of blood) while adjusting for potential confounders, year of study, Dantu and α-Thalassemia genotypes, and anti-malarial drug levels. 4) Least Absolute Shrinkage and Selection Operator (LASSO)^50^, which is a penalised regularization method, was used to determine the minimum number of antigens that account for the highest variance between two outcome groups. LASSO uses a regression model that penalizes the number of variables in the model. Cross-validation analysis was done by sampling with the replacement of 15 variables at a time to identify the model with the minimum lambda value. This model was then used to identify the minimum number of antigens that best discriminated against treated and non-treated volunteers.5) Random forest^25^ was used to select variables in order of importance. A random forest model was fit to the data with bagging. Bagging repeatedly samples from the dataset with replacement and fits decision trees for each sample. The out-of-bag error for each data point was recorded. The random forest model was optimized using a 10-fold cross-validation loop to get the lowest out of bag error and highest accuracy. The best model had 10,000 trees (mtree) with 48 random antigens per tree (mtry). The average error for the forest was analysed, and the importance for each variable was determined by comparing the out-of-bag error before and after permutation over all trees and a score assigned. The score was normalized over the standard deviation, and the variable with the highest score was ranked as the most important. All antigens with a mean decrease accuracy above an arbitrary cut-off of 10% were identified as important in differentiating between treated and nontreated volunteers. All analyses were adjusted for multiple comparisons as appropriate The results of the four antigen selection methods were summarized in a Venn diagram (http://bioinformatics.psb.ugent.be/webtools/Venn/) to identify antigens selected by multiple selection methods.

Analysis of all possible combinations of two of the selected top antigens was determined. The relative risk of developing parasite densities >500 parasites/μl of blood and/or clinical symptoms post CHMI were compared between individuals with high antibody levels and individuals with low to moderate responses for each combination using a modified Poisson regression model. High vs low to moderate antibody level were determined by ranking responses into tertiles and assigning responses in the top tertiles as high and rest as low to moderate. Each combination’s protective efficacy ((1-RR) x 100) was then calculated. This analysis was then repeated for all possible combinations of three, four, five or six of the top antigens. The number of antigens against which a volunteer had high responses to (breadth score) was then assigned.

### Reporting summary

Further information on research design is available in the Nature Portfolio Reporting Summary linked to this article.

## Supplementary information

Supplementary Information

Peer Review file

Reporting Summary

## Source data

Source Data

## Data Availability

All data supporting the findings of this study are available within the paper and its Supplementary Information. Source data are provided with this paper.

## Code availability

All analyses used preassigned code defined within software packages that are publicly available as described in Methods. Any other requests for clarifications may be sought from the corresponding authors.
