# Supplementary material for: Controlled human malaria infection in adults identify combinations of merozoite antigens associated with clinical immunity
Source: Nat Commun. 2026 May 5;17:6472. doi: 10.1038/s41467-026-72716-x (PMC13376759; doi:10.1038/s41467-026-72716-x)
Supplement: Supplementary file 1 — Supplementary Information [file 41467_2026_72716_MOESM1_ESM.pdf]

Table S1: Summary of antigens analysed

	Gene name	Other name	Region expressed	Allele	Expression system	References
**1**	PF3D7_1105200	WRAP73	Full-length ectodomain	3D7	Mammalian	Kamuyu 2017, PhD Thesis
**2**	PF3D7_1455300	null	Full-length ectodomain	3D7	Mammalian	Kamuyu 2017, PhD Thesis
**3**	PF3D7_0423400	AARP	Full-length ectodomain	3D7	Mammalian	Crosnier et al, 2013
**4**	PF3D7_1133400	AMA1	Full-length ectodomain	3D7	Mammalian	Crosnier et al, 2013
**5**	PF3D7_0405900	ASP	Full-length ectodomain	3D7	Mammalian	Crosnier et al, 2013
**6**	PF3D7_0302200	CLAG3.2	Full-length ectodomain	3D7	Mammalian	Crosnier et al, 2013
**7**	PF3D7_1301600	EBA140	Full-length ectodomain	3D7	Mammalian	Crosnier et al, 2013
**8**	PF3D7_0731500	EBA175	Full-length ectodomain	3D7	Mammalian	Crosnier et al, 2013
**9**	PF3D7_0102500	EBA181	Full-length ectodomain	3D7	Mammalian	Crosnier et al, 2013
**10**	PF3D7_1033200	ETRAMP10.2	Full-length ectodomain	3D7	Mammalian	Crosnier et al, 2013
**11**	PF3D7_0828800	GAMA	Full-length ectodomain	3D7	Mammalian	Crosnier et al, 2013
**12**	PF3D7_0930300	MSP1	Full-length ectodomain	3D7	Mammalian	Crosnier et al, 2013
**13**	PF3D7_0206800	MSP2	Full-length ectodomain	3D7	Mammalian	Crosnier et al, 2013
**14**	PF3D7_1035400	MSP3	Full-length ectodomain	3D7	Mammalian	Crosnier et al, 2013
**15**	PF3D7_0620400	MSP10	Full-length ectodomain	3D7	Mammalian	Crosnier et al, 2013
**16**	PF3D7_1036000	MSP11/H103	Full-length ectodomain	3D7	Mammalian	Crosnier et al, 2013
**17**	PF3D7_1035800	MSP3.5/PF10-0350	Full-length ectodomain	3D7	Mammalian	Zenonos et al, 2014
**18**	PF3D7_1035900	MSP3.6/PF10-0351	Full-length ectodomain	3D7	Mammalian	Zenonos et al, 2014
**19**	PF3D7_0207000	MSP4	Full-length ectodomain	3D7	Mammalian	Crosnier et al, 2013
**20**	PF3D7_0206900	MSP5	Full-length ectodomain	3D7	Mammalian	Crosnier et al, 2013
**21**	PF3D7_1035500	MSP6	Full-length ectodomain	3D7	Mammalian	Crosnier et al, 2013
**22**	PF3D7_1335100	MSP7	Full-length ectodomain	3D7	Mammalian	Crosnier et al, 2013
**23**	PF3D7_0502400	MSP8	Full-length ectodomain	3D7	Mammalian	Zenonos et al, 2014
**24**	PF3D7_1228600	MSP9/ABRA	Full-length ectodomain	3D7	Mammalian	Crosnier et al, 2013
**25**	PF3D7_1334600	MSRP3	Full-length ectodomain	3D7	Mammalian	Crosnier et al, 2013
**26**	PF3D7_1334400	MSRP4	Full-length ectodomain	3D7	Mammalian	Zenonos et al, 2014
**27**	PF3D7_1334300	MSRP5	Full-length ectodomain	3D7	Mammalian	Zenonos et al, 2014
**28**	PF3D7_1028700	MTRAP	Full-length ectodomain	3D7	Mammalian	Crosnier et al, 2013
**29**	PF3D7_0612700	P12	Full-length ectodomain	3D7	Mammalian	Crosnier et al, 2013
**30**	PF3D7_0419700	P34	Full-length ectodomain	3D7	Mammalian	Crosnier et al, 2013
**31**	PF3D7_0508000	P38	Full-length ectodomain	3D7	Mammalian	Crosnier et al, 2013
**32**	PF3D7_0404900	P41	Full-length ectodomain	3D7	Mammalian	Crosnier et al, 2013
**33**	PF3D7_1017100	PF10_0166/RON12	Full-length ectodomain	3D7	Mammalian	Zenonos et al, 2014
**34**	PF3D7_1420700	PF113	Full-length ectodomain	3D7	Mammalian	Crosnier et al, 2013
**35**	PF3D7_1136200	null	Full-length ectodomain	3D7	Mammalian	Crosnier et al, 2013
**36**	PF3D7_0606800	VFT1	Full-length ectodomain	3D7	Mammalian	Crosnier et al, 2013
**37**	PF3D7_1137300	null	largest predicted extracellular loop	3D7	Mammalian	Kamuyu 2017, PhD Thesis
**38**	PF3D7_0206200	PAT	largest predicted extracellular loop	3D7	Mammalian	Kamuyu 2017, PhD Thesis
**39**	PF3D7_0525800	IMC1g	Full-length ectodomain	3D7	Mammalian	Kamuyu 2017, PhD Thesis
**40**	PF3D7_0730800.2	null	Full-length ectodomain	3D7	Mammalian	Kamuyu 2017, PhD Thesis
**41**	PF3D7_0830500	TryThrA	Full-length ectodomain	3D7	Mammalian	Kamuyu 2017, PhD Thesis
**42**	PF3D7_0831400	null	Full-length ectodomain	3D7	Mammalian	Kamuyu 2017, PhD Thesis

**43**	PF3D7_0925900	PV5	Full-length ectodomain	3D7	Mammalian	Kamuyu 2017, PhD Thesis
**44**	PF3D7_1025300	null	Full-length ectodomain	3D7	Mammalian	Kamuyu 2017, PhD Thesis
**45**	PF3D7_1229300	null	Largest predicted extracellular loop	3D7	Mammalian	Kamuyu 2017, PhD Thesis
**46**	PF3D7_1237900	null	Full-length ectodomain	3D7	Mammalian	Kamuyu 2017, PhD Thesis
**47**	PF3D7_1252300	null	Largest predicted extracellular loop	3D7	Mammalian	Kamuyu 2017, PhD Thesis
**48**	PF3D7_1345100	TRX2	Full-length ectodomain	3D7	Mammalian	Kamuyu 2017, PhD Thesis
**49**	PF3D7_1401600	PHISTb	largest predicted extracellular loop	3D7	Mammalian	Kamuyu 2017, PhD Thesis
**50**	PF3D7_1460600	ISP3	Full-length ectodomain	3D7	Mammalian	Kamuyu 2017, PhD Thesis
**51**	PF3D7_1462300	null	Largest predicted extracellular loop	3D7	Mammalian	Kamuyu 2017, PhD Thesis
**52**	PF3D7_1364100	PF92	Full-length ectodomain	3D7	Mammalian	Crosnier et al, 2013
**53**	PF3D7_0102700	PFA0135W/Ma TrA	Full-length ectodomain	3D7	Mammalian	Zenonos et al, 2014
**54**	PF3D7_0104200	PFA210c	Full-length ectodomain	3D7	Mammalian	Zenonos et al, 2014
**55**	PF3D7_1021800	PFSEA1	Protein fragment	3D7	Mammalian	Raj et al, 2014
**56**	PF3D7_1436300	PTEX150	Full-length ectodomain	3D7	Mammalian	Crosnier et al, 2013
**57**	PF3D7_0707300	RAMA	Full-length ectodomain	3D7	Mammalian	Zenonos et al, 2014
**58**	PF3D7_0402300	RH1	Full-length ectodomain	3D7	Mammalian	Crosnier et al, 2013
**59**	PF3D7_0424100	RH5	Full-length ectodomain	3D7	Mammalian	Crosnier et al, 2013
**60**	PF3D7_0905400	RHOPH3	Full-length ectodomain	3D7	Mammalian	Crosnier et al, 2013
**61**	PF3D7_0323400	RIPR	Full-length ectodomain	3D7	Mammalian	Zenonos et al, 2014
**62**	PF3D7_0207900	SERA2	Full-length ectodomain	3D7	Mammalian	Zenonos et al, 2014
**63**	PF3D7_0207800	SERA3	Full-length ectodomain	3D7	Mammalian	Zenonos et al, 2014
**64**	PF3D7_0207700	SERA4	Full-length ectodomain	3D7	Mammalian	Zenonos et al, 2014
**65**	PF3D7_0207400	SERA7	Full-length ectodomain	3D7	Mammalian	Zenonos et al, 2014
**66**	PF3D7_0902800	SERA9	Full-length ectodomain	3D7	Mammalian	Zenonos et al, 2014
**67**	PF3D7_0212600	SPATR	Full-length ectodomain	3D7	Mammalian	Crosnier et al, 2013
**68**	PF3D7_0616500	TLP	Full-length ectodomain	3D7	Mammalian	Crosnier et al, 2013
**69**	PF3D7_0629500	AAT1, Segment 1 (SEG1)	Predicted extracellular loop	3D7	Mammalian	Kamuyu 2017, PhD Thesis
**70**	PF3D7_0629500	Segment 2 (SEG2)	Predicted extracellular loop	3D7	Mammalian	Kamuyu 2017, PhD Thesis

All proteins were tagged with ratCD4d3+4,biotinylation sequence,6XHIS on the C-terminal end of the construct on backbone

Table S2 : Antigens selected in order of importance using different analysis approaches.

Order of importance	Wilcoxon rank sum test with BH correction	Cox regression	LASSO	Random forest
**1**	MSP11	MSP11	MSP1	MSP1
**2**	MSRP4	MSP1	MSP11	PTEX150
**3**	MSP1	MSP7	MSP7	PF3D7_1401600 (PHISTB)
**4**	PF3D7_1252300	PTEX150	RAMA	MSP7
**5**	MSP9	PF3D7_1401600 (PHISTB)	PF3D7_1401600 (PHISTB)	PF3D7_0831400
**6**	RAMA	RAMA	PTEX150	RAMA
**7**	P113	MSP9		PF3D7_0206200
**8**	MSP6	PF3D7_1252300		MSP11
**9**	PTEX150	PF3D7_1460600 (ISP3)		PF3D7_1345100
**10**	MSP7	MSP5		PF3D7_1252300
**11**	SERA9	PF3D7_1345100		MSP5
**12**	PF3D7_1401600 (PHISTB)	MSRP4		MSP9
**13**	PF3D7_1136200	PF3D7_0525800 (IMC1G)		PF3D7_1237900
**14**	MSP5	PF3D7_1237900		P41
**15**	PF3D7_0831400	GAMA		PF3D7_0629500_SEG2
**16**	MSP3.5	PF3D7_1136200		PF3D7_1460600 (ISP3)
**17**	PF3D7_1460600 (ISP3)	PF3D7_1462300		
**18**	PF3D7_1345100	PF3D7_0830500		
**19**	PF3D7_1025300	PF3D7_0831400		
**20**	EBA181	PF3D7_0606800		
**21**	PF3D7_0206200			
**22**	MTRAP			
**23**	SERA4			
**24**	PFA210c			
**25**	P41			
**26**	PF10_0166			
**27**	PF3D7_1237900			

BH; Benjamini-Hochberg, LASSO; least absolute shrinkage and selection operator.

Table S3 : Genetic variation of top 33 antigens selected from different analysis methods.

Gene ID	Product Description	Gene Name or Symbol	MIS	MFS	Nsyn SNPs	Syn SNPs	NSyn/Syn SNP Ratio	Total SNPs All Strains	CDS Length	NonSynonymous SNPs per coding length	Protein Length,aa	MW,Da	# TM Domains	SignalP Peptide
PF3D7_0102500	erythrocyte binding antigen-181	EBA181	0.999	-2.102	66	55	1.2	369	4704	0.014031	1567	181150	2	Yes
PF3D7_0104200	StAR-related lipid transfer protein	N/A	0.28	-3.379	25	25	1	222	1401	0.017844	466	53626	0	No
PF3D7_0206200	pantothenate transporter	PAT	1	-2.212	4	11	0.36	138	1698	0.002356	565	62662	11	No
PF3D7_0206900	merozoite surface protein 5	MSP5	0.988	-2.098	6	8	0.75	115	819	0.007326	272	30974	2	No
PF3D7_0207700	serine repeat antigen 4	SERA4	1	0.947	66	87	0.76	209	2889	0.022845	962	108681	0	Yes
PF3D7_0404900	6-cysteine protein P41	P41	0.799	-3.133	12	6	2	174	1137	0.010554	378	43088	0	Yes
PF3D7_0525800	inner membrane complex protein 1g	IMC1g	0.244	-2.655	5	13	0.38	99	903	0.005537	300	34341	0	No
PF3D7_0606800	VFT protein	VFT1	0.124	-2.931	1	14	0.07	133	900	0.001111	299	34503	0	Yes
PF3D7_0629500	amino acid transporter AAT1	AAT1	0.143	-2.882	7	14	0.5	162	1821	0.003844	606	68858	9	No
PF3D7_0707300	rhoptry-associated membrane antigen	RAMA	1	-1.474	77	46	1.67	379	2586	0.029776	861	103641	0	Yes
PF3D7_0828800	GPI-anchored micronemal antigen	GAMA	0.183	-3.301	38	67	0.57	222	2217	0.01714	738	85250	2	Yes
PF3D7_0830500	sporozoite and liver stage tryptophan-rich protein, putative	TryThrA	0.223	-2.668	56	63	0.89	220	2028	0.027613	675	80443	1	No
PF3D7_0831400	Plasmodium exported protein, unknown function	N/A	0.969	-0.655	27	14	1.93	142	909	0.029703	302	36413	1	Yes
PF3D7_0902800	serine repeat antigen 9	SERA9	1	0.045	27	26	1.04	141	2799	0.009646	932	105544	0	Yes
PF3D7_0930300	merozoite surface protein 1	MSP1	0.141	-3.197	312	87	3.59	495	5163	0.06043	1720	195726	1	Yes
PF3D7_1017100	rhoptry neck protein 12	RON12	0.13	-2.976	4	9	0.44	179	933	0.004287	310	36405	1	Yes
PF3D7_1025300	conserved protein, unknown function	N/A	0.121	-3.037	23	23	1	83	2769	0.008306	922	109073	0	Yes
PF3D7_1028700	merozoite TRAP-like protein	MTRAP	1	-1.824	36	25	1.44	241	1497	0.024048	498	58085	1	Yes
PF3D7_1035500	merozoite surface protein 6	MSP6	0.565	-2.477	32	25	1.28	105	1116	0.028674	371	42275	0	No
PF3D7_1035800	probable protein, unknown function	M712	1	-2.143	73	136	0.54	249	2139	0.034128	712	82071	0	Yes
PF3D7_1036000	merozoite surface protein 11	MSP11	0.391	-2.514	9	4	2.25	75	1218	0.007389	405	46729	0	Yes
PF3D7_1136200	asparagine-rich merozoite protein ARMA	ARMA	1	-2.026	44	46	0.96	171	2040	0.021569	679	76570	0	No
PF3D7_1228600	merozoite surface protein 9	MSP9	0.39	-3.212	41	50	0.82	118	2232	0.018369	743	86623	0	Yes
PF3D7_1237900	conserved Plasmodium protein, unknown function	N/A	1	-0.82	132	179	0.74	372	3567	0.037006	1188	140939	1	No
PF3D7_1252300	conserved Plasmodium protein, unknown function	N/A	1	-1.546	2	0	0	155	258	0.007752	85	9443	1	No

PF3D7_1334400	MSP7-like protein	MSRP4	0.12	-3.036	17	4	4.25	21	930	0.01828	309	36383	0	Yes
PF3D7_1335100	merozoite surface protein 7	MSP7	0.459	-2.577	29	10	2.9	150	1056	0.027462	351	41276	0	Yes
PF3D7_1345100	thioredoxin 2	TRX2	0.122	-3.166	4	4	1	54	474	0.008439	157	18630	0	Yes
PF3D7_1401600	Plasmodium exported protein (PHISTb), unknown function	N/A	1	-1.412	19	13	1.46	219	1437	0.013222	478	55827	1	No
PF3D7_1420700	surface protein P113	P113	0.991	0.436	32	37	0.86	222	2910	0.010997	969	112574	1	Yes
PF3D7_1436300	translocon component PTEX150	PTEX150	1	-2.182	36	26	1.38	273	2982	0.012072	993	112409	0	Yes
PF3D7_1460600	inner membrane complex sub-compartment protein 3	ISP3	0.567	-2.264	1	4	0.25	92	447	0.002237	148	17117	0	No
PF3D7_1462300	GTP-binding protein, putative	N/A	0.178	-3.307	27	62	0.44	144	4074	0.006627	1357	161219	3	Yes
PF3D7_0424100	reticulocyte binding protein homologue 5	RH5	0.126	-3.141	16	22	0.73	95	1581	0.01012	526	62996	0	No

Data obtained from PlasmoDB. The mutagenesis data was obtained by piggyBac insertion saturation mutagenesis (28). MIS (Mutagenesis index score): rates the potential mutability of *P. falciparum* genes, <0.5 = likely essential, >0.5 =Likely nonessential. MFS (mutant fitness score): estimates the relative growth fitness cost for mutating a gene, < -2=high fitness cost, > -2=low fitness cost. The genetic variation data is from analysis of 218 reference *P. falciparum* sequences from different geographical regions. SNPs=single nucleotide polymorphisms, NS=Nonsynonymous, S=Synonymous. The ratio of Nonsynonymous to synonymous SNPs is an indicator of selection pressure. The higher the value the stronger the selection pressure. aa=amino acids, MW=Molecular weight, TM=transmembrane domain. The presence of a signal sequence was predicted using hidden Markov model and neutral network prediction tools. Data on Rh5 are included for comparison

Table S4: Baseline characteristics

	Cohort			Total
**Year of recruitment**	**2016**	**2017**	**2018**	
**Sample size**	36	53	53	142
**Age: median (range)**	29 (18-44)	25 (20-44)	27 (18-45)	27 (18-45)
**Sec: Percentage male**	75%	73.6%	60.4%	69%
**No detectable lumefantrine**	15 (41.6%)	28 (52.8%)	31 (58.5%)	74 (52.1%)
**No detectable levels of any of the tested drugs[a]**	13 (36.1%)	23 (43.4%)	28 (52.8%)	64 (45.1%)

[a] The following antimalarial drugs were measured in plasma samples collected on day 7 post challenge: Lumefantrine, Pyrimethamine, Sulfadoxine, Chloroquine, Artesunate and Artemether. Of the 142 volunteers originally included in the study, 78 had detectable Lumefantrine or Sulfadoxine or both at levels below the minimum inhibitory concentrations (MIC, Lumefantrine 200ng/ml, Sulfadoxine 100ng/ml).

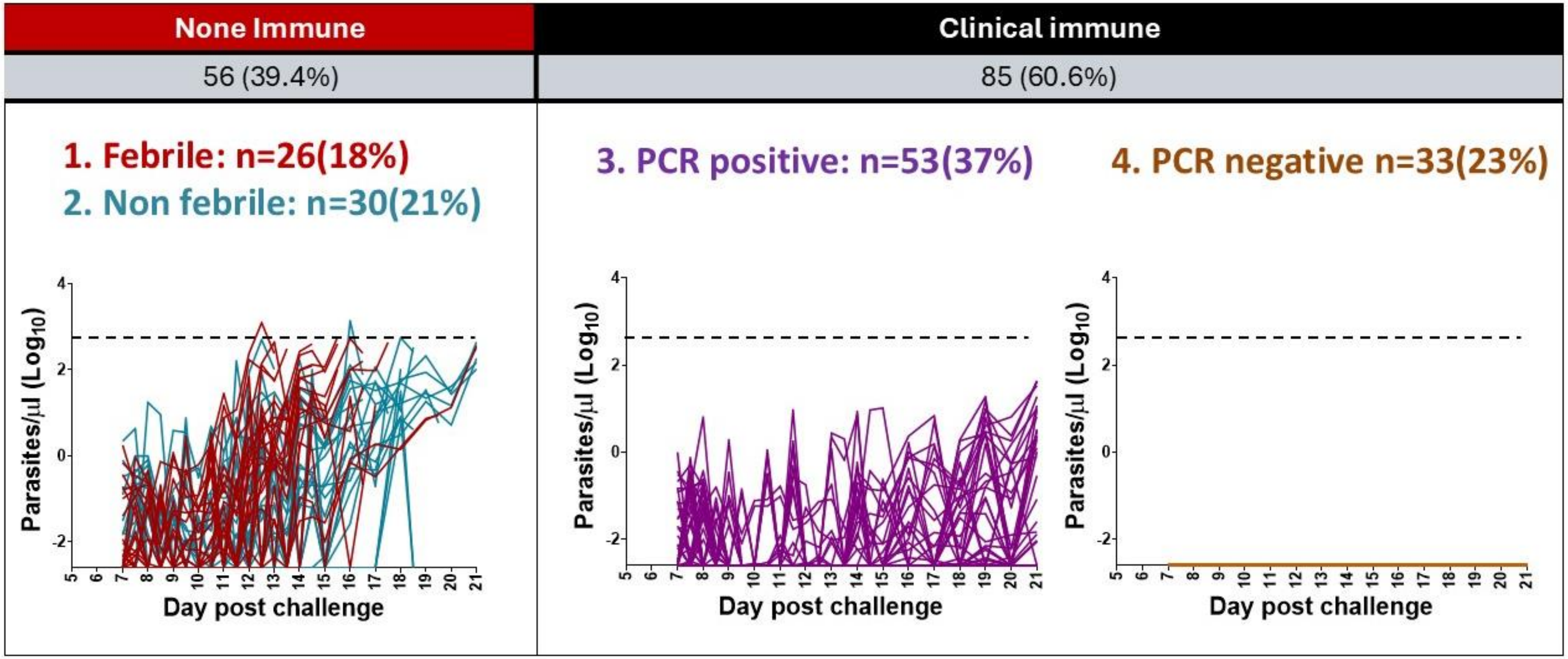

Fig S1: Paraste growth curves: CI volunteers (black); remained afebrile with parasitaemia < 500/µL, n = 86. NI volunteers (red); developed parasitaemia > 500/µL of blood and/or developed fever (≥37.5°C) with any parasitaemia, n = 56. The dotted line indicates the parasitemia treatment threshold (500/µL). The dotted line shows the threshold for treatment.

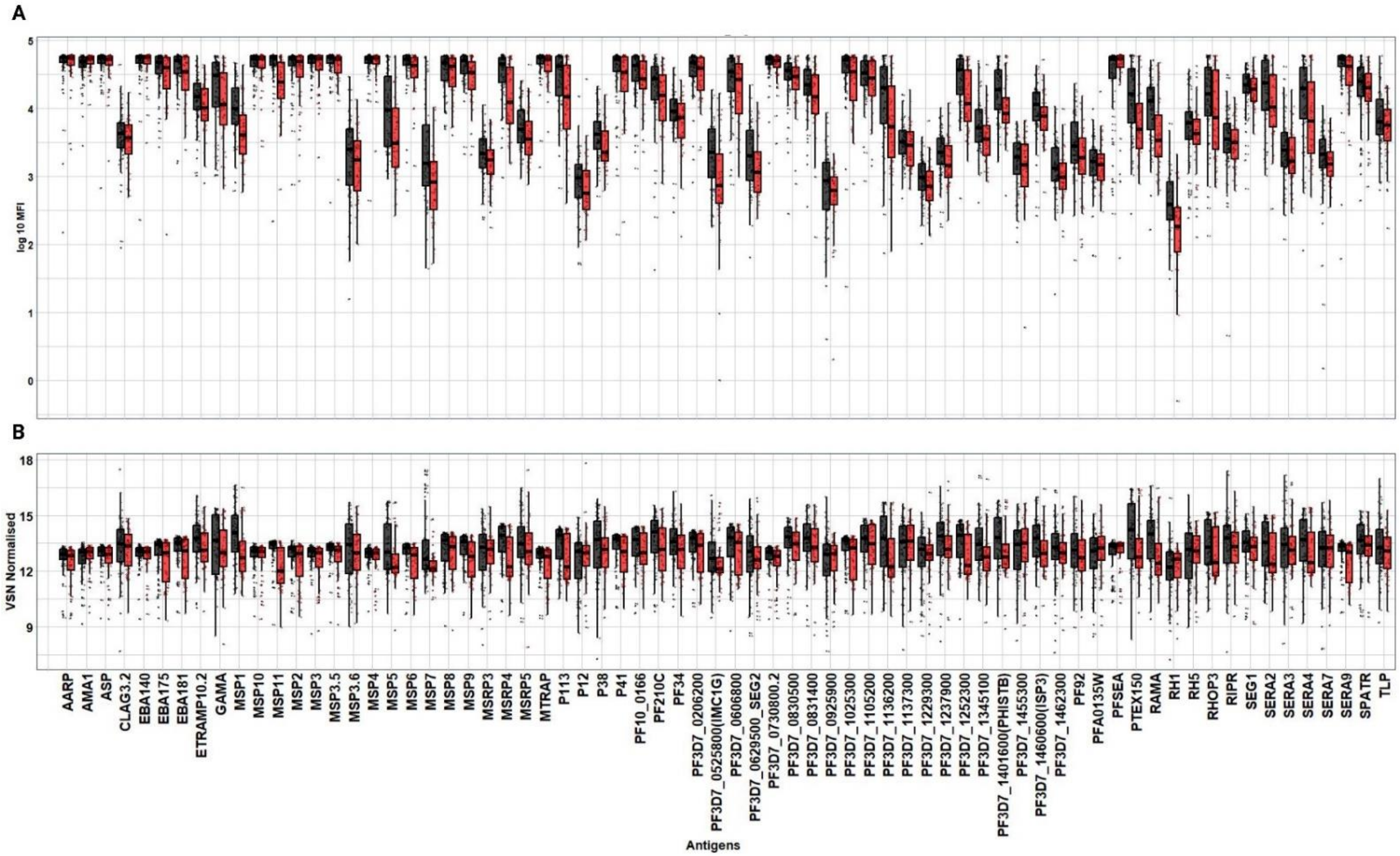

Fig S2: Relative antibody levels for the different antigens following normalization approaches: We show the distribution of responses responses following normalization by **A)** Log10 transformation and **B)** variance stabilization normalization method (VSN). We previously explored four (VSN, Log2, robust-linear-model (RLM) and cyclic loess) functions for normalization to identify one that optimally reduced the mean–variance dependence (MVD) (Mwai k., et al 2021). The VSN was adopted for analyses as it overcomes the limitations of log transformations minimizing the inflated variance around low signal intensities, but also results in normalization as effectively as log-transformation. It calibrates between-feature variation through shifting and scaling mechanism in which all the data are adjusted. Huber et al. and Durbin et al. independently proposed the VSN approach for such data which is a variant of the log-transform (*glog2*). categorized by clinically immune (CI, black), non-immune (NI, red)

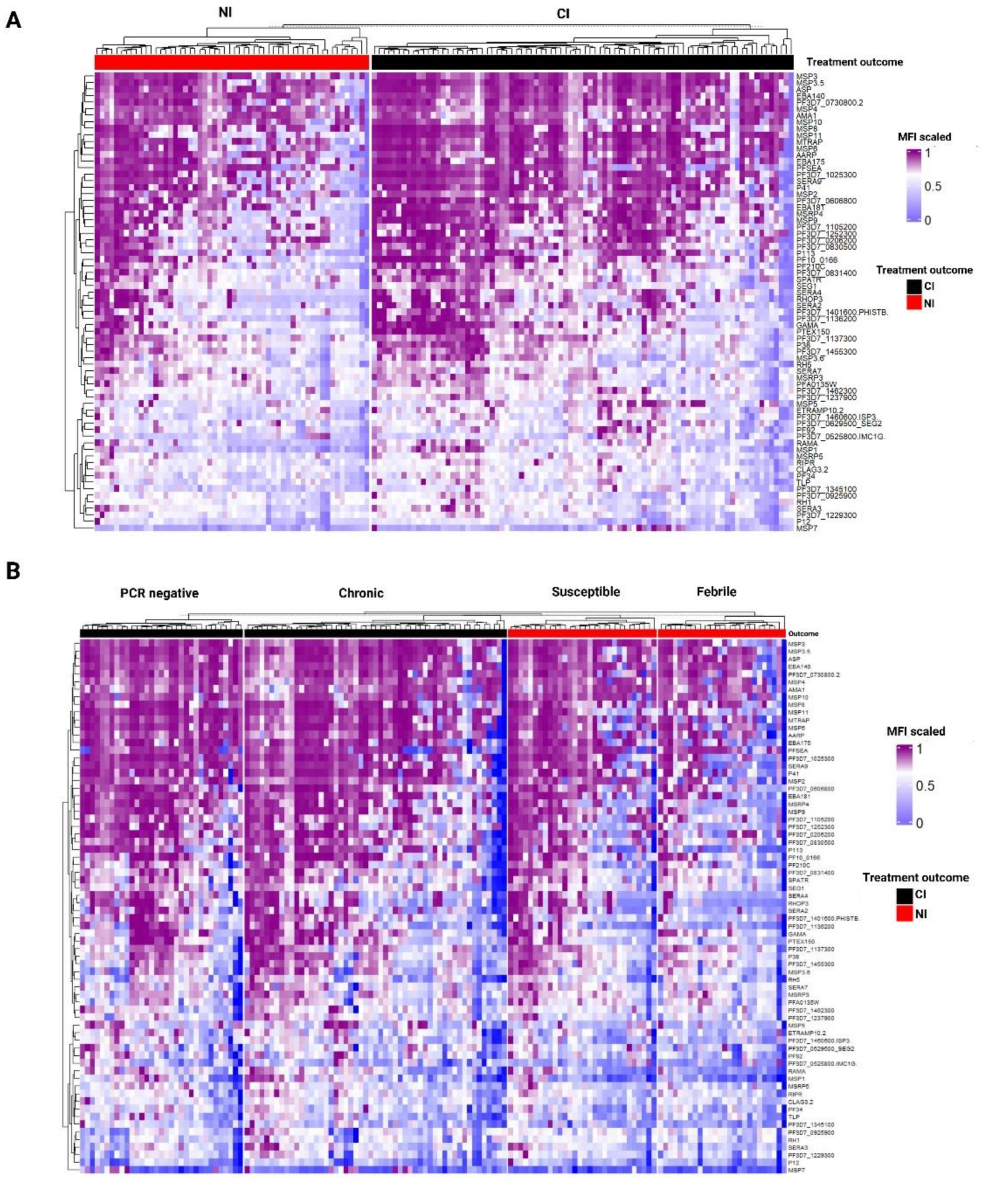

Fig S3: Heat maps showing relative antibody levels A) Antibody responses categorized by clinically immune (CI, black), non-immune (NI, red) B) Antibody reponses the none immune subdivided into those that became febrile and susceptible (those in whom parasite grew above the threshold for treatment (500parasites/µl)) and clinically immune into participants that either maintained parasites below the threshold and those that were PCR negative through 21 days of follow-up.

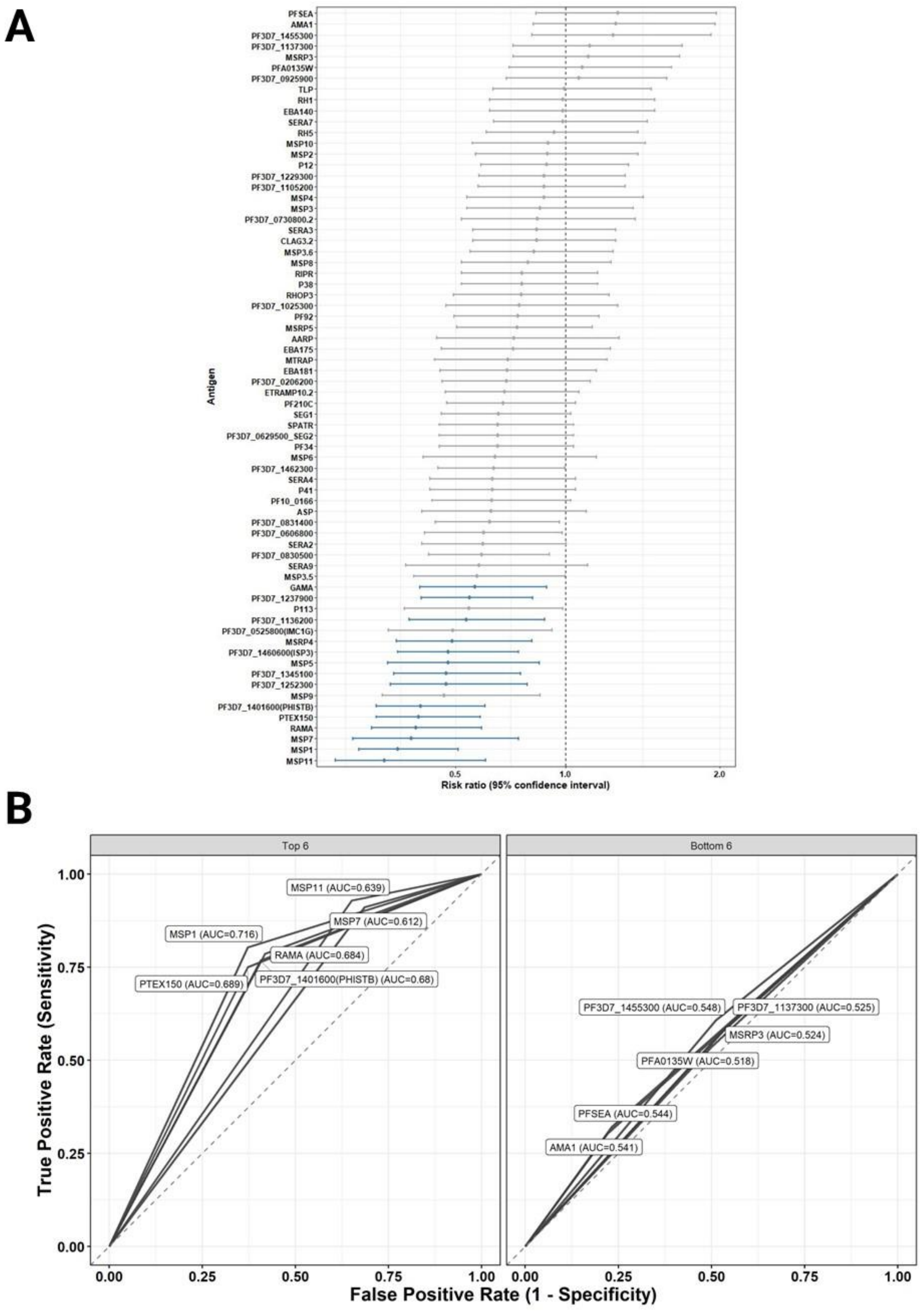

Fig S4: A) Forest plot of Poisson regression point estimates and confidence intervals in 142 participants. The black dotted line represents a hazard ratio (HR) of 1. The dots represent the point estimate for antibody responses against each antigen. The lines illustrate the 95% confidence intervals. HR(95%CI) in blue represent P values that remained significant after Benjamini-Hochberg correction for multiple comparisons.B) ROC curve of Poission regression showing the sensitivity and specificity of CI clacification for the top six antigens and lowest six antigen. Analysis includes n=142 volunters.

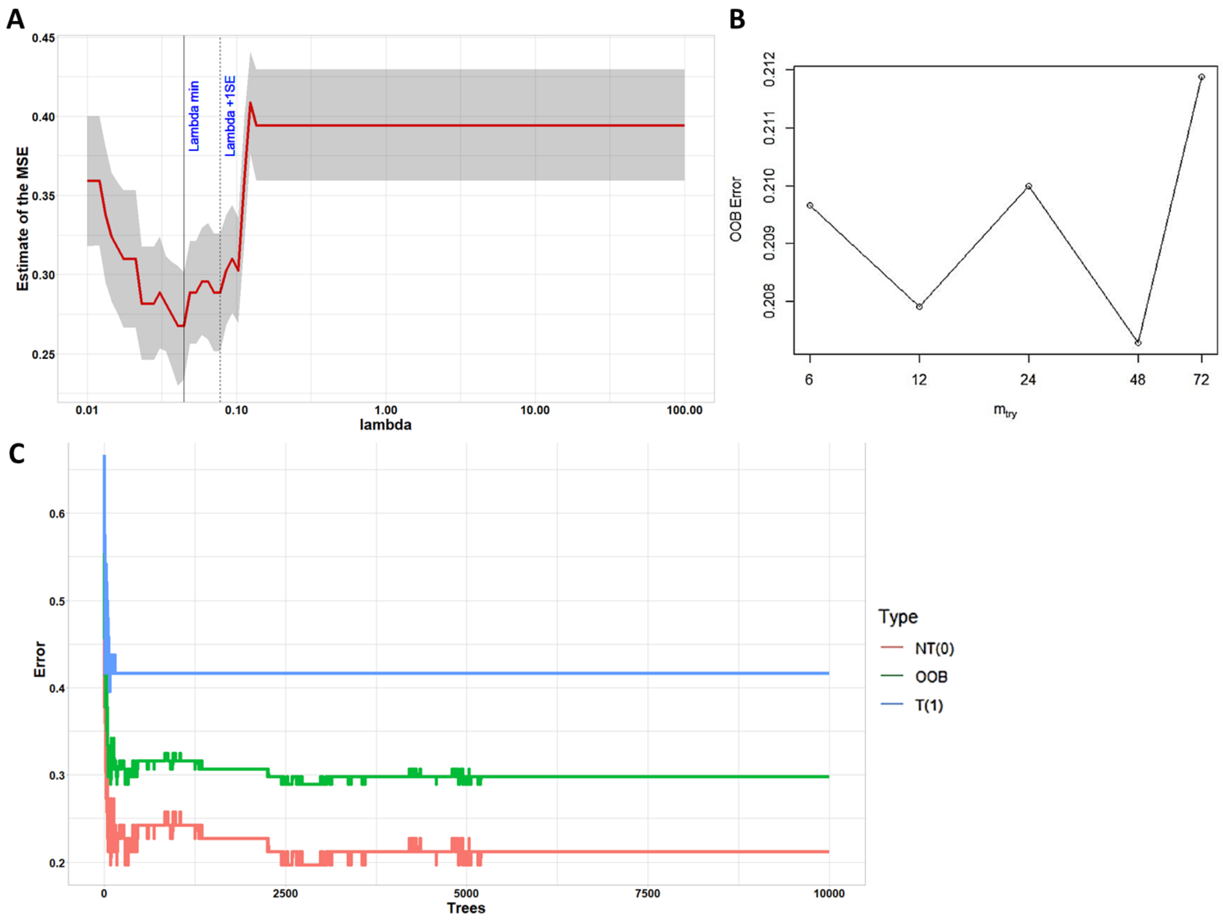

Fig S5: LASSO and random forest model fine tuning

Model tuning parameters were first selected before using the models for antigen selection. **A)** LASSO fine tuning was done using cross-validation analysis. Sampling with replacement of 15 variables at a time was done to find the lambda that minimizes the out-of-sample MSE. The best lambda is the one that produces the smallest out-of-sample MSE. The graph shows MSE for different lambda values, the lowest lambda (lambda min) is indicated. A model with the parameters for the lambda min was used to select the minimum number of antigens that best discriminate between treated and non-treated volunteers. **B)** Random forest model fine tuning was done through bagging. Bagging repeatedly samples from the dataset with replacement and fits decision trees for each sample. The out-of-bag error for each data point was recorded. Random forest model was optimized using a 10-fold cross validation loop to get the lowest out of bag error and highest accuracy. The graph shows the out of bag error levels for different mtry values. The best model had 48 random antigens per tree (mtry). **C**) The graph shows random forest model out of bag error and error in predicting not treated versus treated outcomes using different numbers of decision trees. We chose 10,000 trees (ntree) at which point all the three errors had stabilized. The importance for each variable was determined by comparing the out-of-bag error before and after permutation over all trees and a score assigned. MSE=Mean squared error, NT=not treated, T=treated, OOB=out of bag

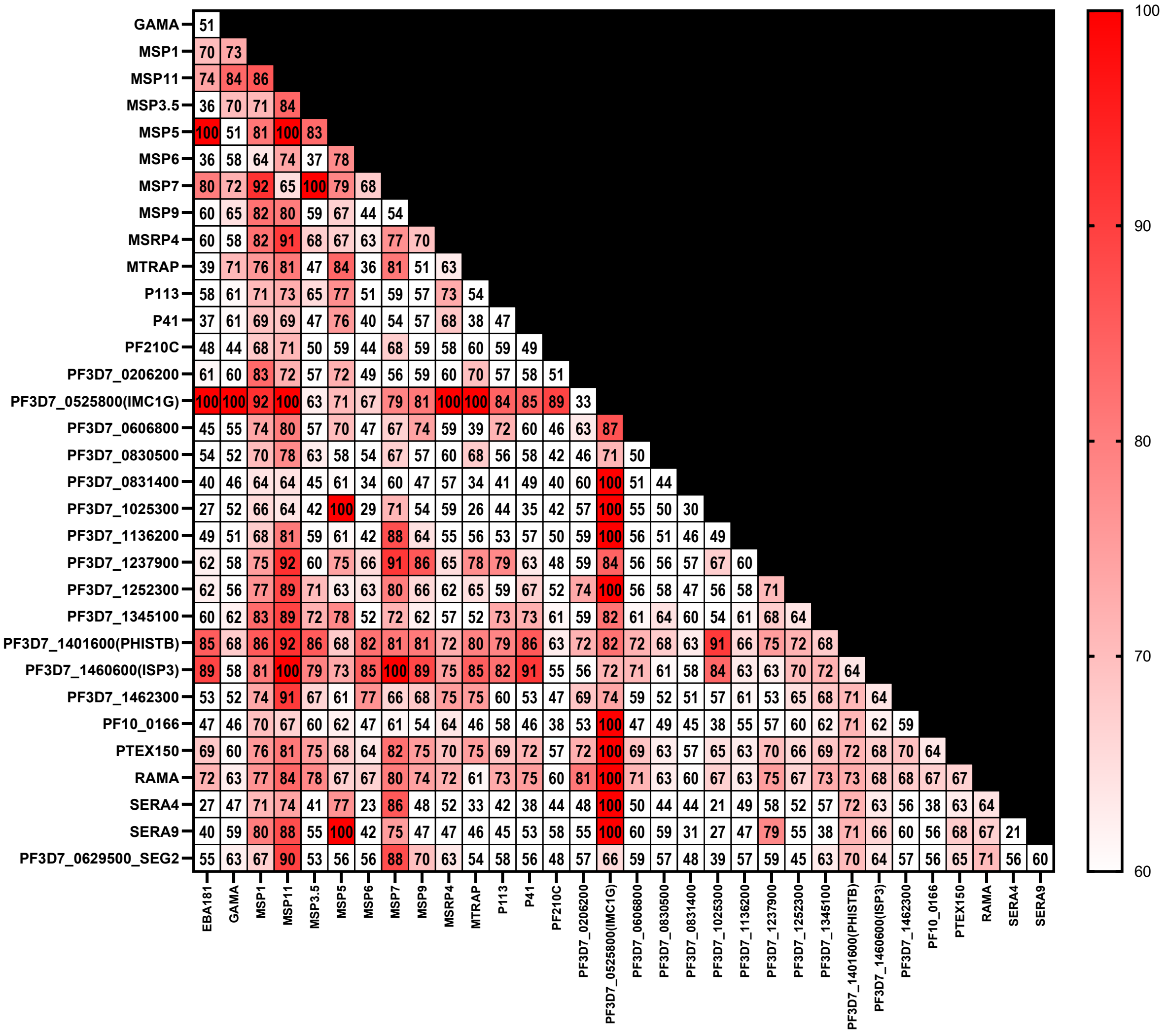

Fig S6: Matrix showing protective efficacy ((1-RR)*100) for different antigen combinations. Boxes in red show combinations with a protective efficacy of ≥60 while all boxes in white show combinations with a protective efficacy ≤60

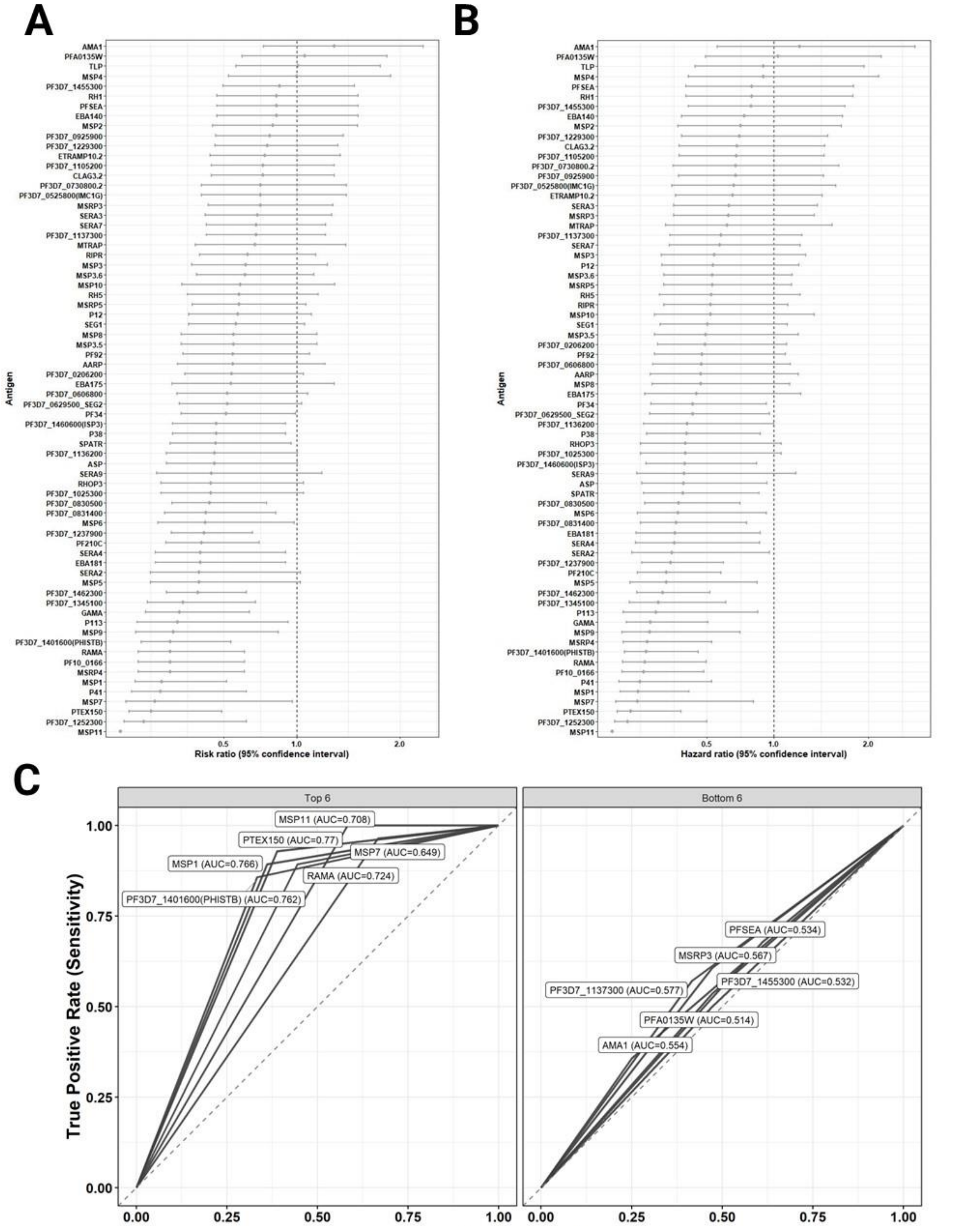

Fig S7: Sensitivity analysis Forest plot of A) Poisson regression point estimates and B) Cox regression Harzard ratios among the subset (n = 64) of volunteers with no detectable serum levels of lumefantrine. Confidence intervals are shown for each antigen. Levels of antibodies to MSP11 perfectly predicted outcome in this sensitivity analysis, confirming its position as a highly significant predictor, and HR and CIs are therefore not shown.C) ROC curve of Poission analysis showing the sensitivity and specificity of CI clacification for the top six antigens and lowest six antigen. Analysis includes n = 64 volunters witn no detectable drug levels.
