# Supplementary material for: Controlled human malaria infection in adults identify combinations of merozoite antigens associated with clinical immunity
Source: Nat Commun. 2026 May 5;17:6472. doi: 10.1038/s41467-026-72716-x (PMC13376759; doi:10.1038/s41467-026-72716-x)
Supplement: Supplementary file 2 — Peer Review file [file 41467_2026_72716_MOESM2_ESM.pdf]

nature portfolio

Peer Review File

Controlled human malaria infection in adults identify combinations of merozoite antigens associated with clinical immunity

Corresponding Author: Dr Rodney Ogwang

This file contains all reviewer reports in order by version, followed by all author rebuttals in order by version.

Version 0:

Reviewer comments:

Reviewer #1

(Remarks to the Author)
This manuscript characterizes naturally acquired antibody responses in a cohort of 142 Kenyan adults that underwent CHMI. Of this cohort, 56 individuals developed clinical malaria or parasite levels above 500/ul and as such were considered non-immune, while the other 86 individuals remained symptom free and were considered clinically immune. By comparing antibody profiles against 70 merozoite antigens using several statistical approaches, the authors identified six antigens that together predict clinical immunity in this cohort. They suggest that these antigens may be prioritized for the generation of next generation vaccines.

The strengths of this study are the use of a well controlled CHMI model and a protein array with (most likely) properly folded antigens presenting conformational epitopes to identify protective immune responses. The manuscript reads well.

The major shortcoming of this study is that it identifies antigens that are associated with immunity and may thus be potential vaccine candidates, but that evidence that these are indeed protective targets is lacking. Immune responses against these antigens may correlate with protection, but may not be causally linked and as such it is remains unclear whether the identified antigens are actual vaccine candidates. To overcome this major limitation of this paper, the authors should demonstrate that the identified antigens are indeed targets for functional antibodies. Ideally, they do this by purifying antigen-specific antibodies from protected volunteers and testing whether these are functional in vitro, for instance in a growth inhibitory assay (GIA), or show that sera depleted of (combinations of) antibody specificities loose activity in such functional assay. Alternatively, they could raise antibodies against the identified targets in rodents and test the activity of antibodies and combinations thereof in functional assays. Evidence that the identified targets are causally linked to the observed clinical immunity would make this manuscript much stronger and substantially more relevant for the malaria vaccine research community.

Even if the authors can prove that the six antigens can elicit functional antibodies (or at least a few of them), it is really difficult to see how a combination of six antigens can become a viable vaccine candidate. Not only seems the need to target six candidates for one stage excessive, some are prohibitively large proteins making it hard to produce these, and poorly conserved. The authors should at least discuss how they see that identification of these six antigens can lead to a new blood stage vaccine, providing more details on next steps.

What is interesting is that the authors not only identify a panel of 6 antigens that are together predicted to protect 100% against clinical malaria, they also run an additional analyses (Fig 4a) that shows that combinations of 2 (or 3) may also be 100% protective. This panel would, from a vaccine development point of view, be much more attractive to focus on. Can the authors explain why they think that the panel of 6 antigens is more relevant? It is currently difficult to grasp which of the two results, Fig 4a or Fig 4b, should be given more value. Also, it would be helpful to include a supplementary figure with a 33 x 33 matrix highlighting which combinations of 2 antigens are predicted to give 100% protection.

While one of the strengths of this study lies in the cohort that the authors use, they do not provide much background information about this is cohort. The cohort characteristics and parasitaemia over time may have been published earlier, but it would be very helpful to provide these data here in a supplementary figures so readers do not have to search elsewhere

for this information. The parasitaemia over time, grouped by CI vs NI, would be helpful. Related to this point, were there volunteers that cleared infection and did these volunteers have different immune signatures compared to volunteers that stayed clinically immune but still had substantial parasitaemia?

For vaccine development it is obviously important that the targets are genetically conserved, which has been a challenge in particular for blood stage antigens. Therefore it is valuable that the authors include a genetic analysis of the identified targets. However, this section of the manuscript requires substantial attention. dNS/dS values are indeed a good indication for immune pressure, but when discussing overall conservation, it is more meaningful to look at non-synonymous SNPs per coding sequence length. Also, values for NS/S SNP ratio's in table S3 do not seem to be correct (assuming that # NS SNPs and total SNPs are correct). E.g. only one quarter of the RH5 SNPS are NS SNPs (22 out of 95) so a value of 11 for NS/S SNP ratio does not seem to make sense.

With limited expertise in statistics, we cannot judge whether the statistical analyses used in this study are methodologically sound and/or appropriately applied, and we therefore recommend that an expert in statistics assesses this aspect of the manuscript.

Other points:
- Figure 2A – There seem to be 71 antigen reactivity rows, rather than 70.
- Figure 2A – Text in top right corner "groups" is not aligned properly
- Figure 2A – Could a supplementary figure be added that shows the same data, but with the immune and non-immune people grouped? This would be helpful to compare responses within groups
- Legend Figure 2A – "Treatment outcomes are annotated in the two rows", there seems to be only one row.
- Figure 3A X axis should be corrected from Not treated-Treated to Clinical Immune- Non Immune.
- Figure 4B is related to Figure 3, and Figure 4A and 4C are related. Therefore it seems sensible to change the order of the figures, either by swapping 4A and 4B or integrating 4B in Figure 3.
- Legend Figure 4A: comparison "between individuals with high versus low-to-moderate antibodies". Define high and low-to-moderate cutoff.
- Legend Figure 4A: correct typo in "Combination analysis the top 33 candidates"
- Specify which version of PlasmoDB has been used for retrieving sequence data to do the sequence analysis.
- Lines 199-200: names of top 6 antigens changed to MSP1, MSP11, MSP5, IMC1G and ISP3, whereas everywhere else they are MSP1, MSP11, RAMA, PTEX150 and PHISTb.
- It is not clear how many antigens have been excluded from analysis. Line 88 says 70 antigens out of 110 were included for analysis, but methods only refer to 25 being excluded, making a total of 85 antigens to include for analysis.

Reviewer #2

(Remarks to the Author)
The authors previously conducted controlled human malaria infection (CHMI) studies in Kenyan adults and demonstrated that clinical immunity was strongly correlated with IgG Fc-mediated functional activity against whole merozoites. In this study, they utilized a KILchip system to identify the merozoite antigens that induce antibodies associated with clinical immunity observed in the CHMI study. Using five distinct methodologies, they consistently identified six key antigens: MSP1, MSP11, RAMA, MSP7, PF3D7_1401600, and PTEX150. Selected combinations of these antigens predicted 100% protection against malaria. Although all the studies are carefully designed and conducted, the primary objective—an examination of the IgG Fc-mediated functional response—was not directly addressed. Additionally, the contribution of the selected antigens to the IgG Fc-mediated functional response has not been experimentally validated. I have comments to improve this manuscript.

Major Comments:
1) Lines 66-68: The authors stated the objective of their study: "We therefore sought to identify additional merozoite antigens that, in combination, would potentially increase the potency of the IgG Fc-mediated functional response." However, they only measured the levels of IgG against 70 Pf antigens using the KILchip system. To meet their objectives, they should assess the Fc-mediated functional response for at least one of the selected antigens beyond MSP1, as they are the leading experts in this research field. This step is crucial for validating their selection results.
2) One of the top six antigens, PTEX150, was selected. However, this protein is well-known as one of the components of PTEX, which is involved in the trafficking of the parasite's proteins to the red blood cell (RBC) cytoplasm. There is no interpretation or discussion regarding the significance of selecting PTEX150 in this study.
3) In this study, "protection" refers to the "protection against IV PfSPZ challenge." Therefore, I believe that including PfCSP as one of the test antigens is essential.
4) To assess the quality of proteins expressed in the KILchip, please include the "expression system" in Table S1 and incorporate this into the analyses. Furthermore, for proteins expressed in Expi293, does their "codon optimization" eliminate N-glycosylation?

Minor comments:
5) Line 40: "drug-resistant parasite" should be mentioned.
6) Line 299: Are there any parasitic individuals at the recruitment? If so, exclude or treat them before the inclusion.

Reviewer #3

(Remarks to the Author)
The study leverages a large human challenge model in semi-immune population to identify IgG responses that account for natural acquired immunity against Plasmodium falciparum clinical episodes. Researchers utilized a custom merozoite protein microarray (KILchip) to screen immune responses in semi-immune Kenyan adults and employed five complementary statistical methods—including traditional models and machine learning approaches—to prioritize antigens associated with clinical immunity. Six antigens (MSP1, MSP11, RAMA, MSP7, PF3D7_1401600 (PHISTB), and PTEX150) were consistently associated with protection across all analyses. Moreover, the use of combinations of antibody responses was more predictive than a single antigen, suggesting that independent contributions to protection may be at play. The study identified antibodies against Pf merozoite antigens that had been both reported as markers of naturally acquired immunity in previous studies and others that had not yet been reported. Unlike observational longitudinal studies of cohorts where exposure is not controlled, results from large CHMI like this present less sources of confounding and are of great value. These findings are important to identify new vaccine candidates

However, this study presents important shortcomings in the reporting and in the methodology:

Major:

1. We miss a figure reporting the original MFI data –after pre-processing, i.e. exclusions and noise subtraction, that feed all subsequent analyses. Original data should be shown to readers in the manuscript, not in the supplementary. Furthermore, such figure would benefit from plotting separately MFI measurements from NI and CI (by colouring or plotting them as separate paired boxplots). This plot partly already exists but is placed in the supplementary (S1).

This Figure S1 raises the following issues:

1.1. There are several well-known markers of exposure (and candidate determinants of protection) that clearly have a saturated signal: AMA1, EBA140, EBA180, MSP2, MSP3, MSP4… This should be acknowledged at least as a limitation in the discussion. In Lines 113 and elsewhere I believe, the authors highlighted they did not find any association with protection for AMA1 or MSP3 as probably expected. Maybe here they have one possible explanation for this. Potential good predictors of immunity contained in the microarray may have been detected as indeed associated with protection, had they been measured in an assay or at a dilution that was able to resolve differences for higher Ab concentrations.

1.2. As expected, Figure S1 shows that the MFI measurements follow a rather log-normal distribution, and thereby log-transformation of MFI measurements seems well-justified (which incidentally was done in the Figure S1) . However, in most analyses in the manuscript, the authors preferred to use natural scale MFI values. For antibody levels/concentrations, researchers usually compare geometric means instead of arithmetic means and use log-transformed values as covariates in their (generalized) linear models. The authors should make clearer in the methods that they indeed do not use log-transformed MFI values and should provide a convincing rationale for this unusual choice.

2. The method to assess the accuracy for predicting clinical immunity using a combination of Ab responses is complicated, not well described and may potentially be too optimistic because it may suffer from overfitting, as the authors do not validate or cross-validate the whole process.

2.1. Lines 413, how are high and low Ab levels determined? I cannot see it described in the methods or elsewhere. Is this determined based on the linear combination of log (MFI) of the different Ab using the coefficients obtained from the modified Poisson regression applied repeatedly on all the studied combinations? If so, what is the threshold for classifying between high and low using this linear combination?
2.2. I do not understand the purpose of fitting models using an increasing number of Abs in one case from the final subset of best predictors and from the total 33 Abs and then compare one procedure with the other. Of course, average prediction scores will be better when using combinations from the subset of best predictors. This is a trivial result
2.3. The formula for obtaining the "protective efficacy" is (1-RR)*100. The authors should clarify where the RR estimates come from. Do they come from the exponentiated modified Poisson regression coefficients of a binary covariate (high/low antibody responder given a combination of Ags?). Please clarify? Be as it may, you should provide confidence intervals for your RR estimates.

2.4. More specifically, reporting RR (or, what is equivalent, efficacy as 1-RR) in this context can be misleading, especially if we do not know which individuals are classified as high and low responders. You may have a small group of so-called high responders that are all CI, whilst the low responders contain both NI and CI. RR of high over low responders will then be 0, yet both the true positive rate and the true negative rate of your Ab-based prediction may be considerably lower than 100%. This may explain the surprising result that whereas you can achieve 100% efficacy with only 2 Ag, the LASSO penalized regression chose 6 regressors. This is because 100% efficacy does not mean 100% accuracy, and the departure from one another can be very large.

In studies assessing the prognostic (or diagnostic) predictive power of biomarkers, one would expect to report the true positive rate (sensitivity) and true negative rates (specificity) of your multi-Ag panel to predict clinical immunity. Providing ROC curves would have been nice, by sliding the threshold separating high and low Ab groups. Additionally, whatever prediction score you choose to report, it should be validated with a validation subset unseen by the algorithm (including the filtering process that the authors used to select the final 6 best predicting Ags) or using cross-validation. In such studies, the greatest danger is always overfitting.

3. Lines 385-391: Why do authors adjust the p-value for multiple comparisons for method 1) but not for method 2) and 3), unless they do, and they didn't specify it in the manuscript (Figure 3A-C)? Modified Poisson and PH Cox regressions are conducted repeatedly for each Ag, therefore they should also be adjusted for multiple comparisons to report findings with a controlled false discovery rate.

4. The study places a lot of emphasis on the use of "independent analytical approaches", implying that converging results will necessarily be robust and method-independent. But the statistical and machine learning methods they used are very different to each other and respond to different objectives. They are necessarily bound to yield different results. These results are not necessarily incorrect because they do not agree with each other. They simply respond to different questions. The authors themselves acknowledge this : "…in focusing on the six antigens that were consistently identified, it is likely that we are excluding antigens that were genuinely associated with outcome but where methodological issues led to erroneous exclusion in one analytical approach". It is not likely, the chosen strategy will necessarily do this.

For instance, whilst the univariate methods: 1, 2 and 3 are intended to detect associations with protection with a given false discovery rate, and, to a certain extent, they should be quite comparable provided p_values are corrected for multiple comparisons in all of them, methods 4 and 5 are of a completely different nature. They simply maximize prediction and will select whichever Ags are best (and not redundant) at achieving it. Furthermore, the lasso-penalised regression will also try to achieve this with as few Ags as possible. Therefore, if a lasso-penalised regression does not select a certain number of Ags, this is by no means evidence that the excluded Ags are not associated with protection. Simply, it means that the algorithm could predict immunity by means of other (probably redundant) responses against other Ags. I can see numerous Ab responses against Ags that are significantly and quite strongly associated with protection using methods 1,2 and 3, but are excluded from the final 6-Ag set for this reason. Is this not giving the misleading idea that they are worse vaccine candidates?

Minor:

1. Line 91: "breadth" is defined as the number of antibody "high responses". Where in the manuscript is a high/moderate/low response defined and a method/threshold to identify them established?
2. Line 99: "magnitude" is defined as the sum of MFIs. It seems you are computing the arithmetic mean, not the geometric mean. This has to do with the fact that you usually use natural scale MFI, not log-transformed MFI. Given the distribution of MFI (see S1), the arithmetic mean will be very much (perhaps too much) driven by the far end of the upper tale.
3. 138-140: The description of what LASSO penalty achieves in a regression is incorrect. LASSO does not minimize variance between outcome groups as stated. Lasso tries to minimize prediction error while keeping the model simple in terms of number of predictors (sparse)
4. Line 142: regarding cross-validation, you said "sampling with replacement of 15 variables at a time". Do you mean 15 individuals at a time?
5. In line 377, authors state that "duplicate spots with a coefficient of variation greater than 20%" were re-assayed. Which duplicates are the authors referring to? Blank controls, protein tag controls or all test samples that were measured in duplicates? Authors should explain this.
6. Lines 376-377: 23 GST- and 2 MBP-tagged antigens were excluded from the microarray because of high signal from the corresponding protein tag control samples. Does this mean authors excluded 25 measurements or more generally they excluded 25 antigens from all multiplexed measurements as I understand they did? Authors should specify the criterion for exclusion in a quantitative way.
7. Line 388. I can understand that a "modified Poisson regression" was used to suit a binary outcome and be able to calculate relative risks from regression coefficients. Please explain this in the methods
8. Lines 391-394. Lasso is not a regression method, but a penalisation (a.k.a. regularisation) that is applied to a given regression. We do not know what type of regression authors are conducting here. Because the outcome is binary, one would expect they used a lasso-regularised logistic or modified Poisson regression. They could also have conducted a lasso-regularised Cox regression. Or, more difficult to justify, a lasso-regularized linear regression. Authors should specify which type of lasso regression they used.
9. Lines 141. It is incorrect to say that the method for choosing the lambda through cross-validation was done through selecting its minimum value as it is stated. It probably was obtained from minimising the value of a certain error score or loss function, e.g. Poisson deviance?
10. Lines 149. "fitting decision trees for each sample, and noting the out-of-bag error for each data point " This description is a little confusing. Here, I presume authors mean that they fit a decision tree for each bag of samples (random subsets of the data sampled with replacement) and noted the out-of-bag error from its prediction performance on the left-out samples. Please clarify.
11. Lines 401: The authors should specify what type of out-of-bag error measure was optimized in the hyperparameter tuning using cross-validation
12. What is the distance metric used in the hierarchical clustering of Figure 2? If it is a Euclidian distance, then whether data was log transformed or not prior to clustering can make a considerable difference. It is odd that the unsupervised algorithm does not cluster according to CI and NI. Do the authors have any explanation?
13. Figure 4C legend. There seems to be a preposition "of" missing

Reviewer #4

(Remarks to the Author)
I co-reviewed this manuscript with one of the reviewers who provided the listed reports. This is part of the Nature Communications initiative to facilitate training in peer review and to provide appropriate recognition for Early Career Researchers who co-review manuscripts.

Version 1:

Reviewer comments:

Reviewer #1

(Remarks to the Author)
I would like to thank the authors for the changes they have made which in my view have improved the manuscript. While it is unfortunate that the revised manuscript still lacks functional data that demonstrates that the identified candidates are causally linked to protection or that at least one of these can induce antibodies with some functional activity, I understand that experiments demonstrating such causality would require substantial time and resources. I appreciate that the authors have changed some of the wording to more accurately reflect the findings and tone down on identification of new vaccine candidates. Further to the proposed changes, I would like to suggest to also change the title accordingly, to put less emphasis on new vaccine candidates and more focus on the actual findings in the current work. I have no further comments.

Reviewer #2

(Remarks to the Author)
All comments have been appropriately addressed, and the revised manuscript has significantly improved.

Reviewer #3

(Remarks to the Author)
I thank the authors for carefully considering my comments. The majority of my concerns were either addressed with additional plots or analyses or, at the very least, discussed and clarified in the manuscript.

However, given that the authors adopted an analytical approach based on assessing CI prediction –no modelling or causal inference framework-- I still don't understand why they didn't show ROC curves for CI classification, providing thus information on sensitivity and specificity of their classification, and, by this means, also reporting quite transparently how good their high vs. low antibody response threshold was, since ROC curves would have involved shifting this.

Additionally, their ROC curve estimates could have been cross-validated by creating different folds of participants, training their predictive models on one set of folds and testing them on the remaining set recursively.

The authors mention their internal cross-validation of the Random Forest; however, this refers to the bootstrap sampling of features and observations (OOB error) used for hyperparameter tuning. This does not replace independent k-fold cross-validation at the participant level, which is necessary to generate unbiased estimates of predictive performance and to justify the 100% protection claim made in the abstract --100% which in immunology sounds inevitably shocking and unheard of, and still more for a pathogen such as malaria for which immunity is, to a great extent, acknowledged as partial.

I leave to the editor whether to push or not for this important methodological requirement. Apart from this, the manuscript has now considerably improved. I wish the authors the best of luck with the reception of their important work and its continuation.

Reviewer #4

(Remarks to the Author)
I co-reviewed this manuscript with one of the reviewers who provided the listed reports. This is part of the Nature Communications initiative to facilitate training in peer review and to provide appropriate recognition for Early Career Researchers who co-review manuscripts.

Open Access This Peer Review File is licensed under a Creative Commons Attribution 4.0 International License, which permits use, sharing, adaptation, distribution and reproduction in any medium or format, as long as you give appropriate credit to the original author(s) and the source, provide a link to the Creative Commons license, and indicate if changes were made.
In cases where reviewers are anonymous, credit should be given to 'Anonymous Referee' and the source.
The images or other third party material in this Peer Review File are included in the article's Creative Commons license, unless indicated otherwise in a credit line to the material. If material is not included in the article's Creative Commons license and your intended use is not permitted by statutory regulation or exceeds the permitted use, you will need to obtain permission directly from the copyright holder.
To view a copy of this license, visit https://creativecommons.org/licenses/by/4.0/

We appreciate all the reviewers for their helpful comments and suggestions. We hope that all the changes made improve the clarity and quality of the manuscript.

Please see below a point-by-point responses to all the comments and suggestions. Page and line numbers are based on the track changed document.

REVIEWER COMMENTS

Reviewer #1 (Remarks to the Author):

Introduction

This manuscript characterizes naturally acquired antibody responses in a cohort of 142 Kenyan adults that underwent CHMI. Of this cohort, 56 individuals developed clinical malaria or parasite levels above 500/ul and as such were considered non-immune, while the other 86 individuals remained symptom free and were considered clinically immune. By comparing antibody profiles against 70 merozoite antigens using several statistical approaches, the authors identified six antigens that together predict clinical immunity in this cohort. They suggest that these antigens may be prioritized for the generation of next generation vaccines.

1.1 Reviewer comments

The strengths of this study are the use of a well-controlled CHMI model and a protein array with (most likely) properly folded antigens presenting conformational epitopes to identify protective immune responses. The manuscript reads well.

Author response

Thank you for this positive review of our work. We previously evaluated the folding of the different proteins on our microarray chip. This was published in our earlier manuscript (Kamuyu G, et al.,2018) and we indeed agree with the reviewer that these proteins are correctly folded.

1.2 Reviewer comments

The major shortcoming of this study is that it identifies antigens that are associated with immunity and may thus be potential vaccine candidates, but that evidence that these are indeed protective targets is lacking. Immune responses against these antigens may correlate with protection, but may not be causally linked and as such it is remains unclear whether the identified antigens are actual vaccine candidates. To overcome this major limitation of this paper, the authors should demonstrate that the identified antigens are indeed targets for functional antibodies. Ideally, they do this by purifying antigen-specific antibodies from protected volunteers and testing whether these are functional in vitro, for instance in a growth inhibitory assay (GIA), or show that sera depleted of (combinations of) antibody specificities loose activity in such functional assay. Alternatively, they could raise antibodies against the identified targets in rodents and test the activity of antibodies and combinations thereof in

functional assays. Evidence that the identified targets are causally linked to the observed clinical immunity would make this manuscript much stronger and substantially more relevant for the malaria vaccine research community.

Author response

We agree that the manuscript does not evaluate functional responses. We have revised the introduction to remove mention of this as an aim. In addition, we have revised the manuscript to more carefully present our findings as associations with protection. A similar concern was raised by reviewer two. We believe that this re-wording represents our findings more accurately.

Please see page 3, line 66 – 68, we have deleted the sentence "*We therefore sought to identify additional merozoite antigens, that in combination would potentially increase the potency of the IgG Fc-mediated functional response*."

We have replaced this with "*We therefore sought to identify merozoite antigens, that may independently and or in combination be associated with greater protection to clinical malari*a, *with the aim of informing further pre-clinical and clinical development"*

In addition, we discuss this as a limitation and the need for data to demonstrate functional capacity of the identified antigens. This work is planned but is a significant undertaking requiring dedicated resources and time.

Please see page 14, line 320-327. We have inserted "*Another limitation is although we identify combinations of antigens associated with protection we do not show whether these specific responses may increase the potency of the IgG Fc-mediated response that we have previously shown important for protection. Indeed, future studies using purified antigen specific IgGs, monoclonal antibodies, or serum depletion experiments are required to investigate the functional potency of these identified antigens* ."

Furthermore, we think that demonstrating functionality of our findings would be a major undertaking. One could propose mouse immunizations, or antibody purification, and then in vitro functional assays, but we do not believe there is a single canonical functional assay that could be used as a definitive answer, and we would need to consider a number of antigens and potentially combinations (see below). This will require substantial further funding and development. We would suggest that taken together, the identification of novel antigen targets with apparent associations with protection, and the identification of potentially synergistic combinations of antigens, is relevant to the malaria vaccine community and will stimulate further lines of research with differing vaccine platforms and experimental approaches.

1.3 Reviewer comments

Even if the authors can prove that the six antigens can elicit functional antibodies (or at least a few of them), it is really difficult to see how a combination of six antigens can become a viable vaccine candidate. Not only seems the need to target six candidates for one stage

excessive, some are prohibitively large proteins making it hard to produce these, and poorly conserved. The authors should at least discuss how they see that identification of these six antigens can lead to a new blood stage vaccine, providing more details on next steps.

Author response

We agree with this concern, and we have clarified the text to make it clear that we are not proposing a six-antigen combination for future clinical development. Rather, we are proposing that among the 70 plausible antigens that we examined, we identify six antigens which would be worth further study. There would be a number of steps in addressing these concerns including pre-clinical vaccinations with different adjuvant and vectors, screening for functional antibodies, examining combinations, and then undertaking challenge studies of vaccinated animals.

The identification of these protective antigens is a first step. Further epitope and structural level characterisation of these antigens may elucidate the critical epitopes required for protection within a multivalent vaccine. We have discussed this further within the discussion.

Please see Page 14, line 301-311, We propose the following additional text:

"Given the complexities of vaccine development, the antigens that we have identified as having potential for combination or multivalent vaccines will need further reduction and refinement. We consider these data as important first steps. Further epitope mapping and structural level characterization will need to be applied to elucidate and subsequently validate the critical epitopes necessary for protection and amenable to vaccine development. Further studies would include vaccination of mice to determine functional immunity and epitope and structural level characterization of these antigens may elucidate the critical epitopes required to mediate protection. Antigens raising non-functional responses would be de-prioritized, and further challenge studies with multiple combinations would identify the minimum number of antigens required for further clinical development. We would prioritize testing combinations of 2 or 3 antigens that were associated with the highest levels of protection in our study"

1.4 Reviewer comments

What is interesting is that the authors not only identify a panel of 6 antigens that are together predicted to protect 100% against clinical malaria, they also run an additional analyses (Fig 4a) that shows that combinations of 2 (or 3) may also be 100% protective. This panel would, from a vaccine development point of view, be much more attractive to focus on. Can the authors explain why they think that the panel of 6 antigens is more relevant? It is currently difficult to grasp which of the two results, Fig 4a or Fig 4b, should be given more value. Also, it would be helpful to include a supplementary figure with a 33 x 33 matrix highlighting which combinations of 2 antigens are predicted to give 100% protection.

Author response

We agree with the reviewer's assessment that from a vaccine viewpoint a combination of 2 or 3 antigens would be most attractive. We do not think that the final design of a vaccine for clinical development is possible from the data presented alone, rather that further optimization of combinations will be required from additional pre-clinical studies as

mentioned in our response above (comment 1.3). We think that both figure 4a and b are useful to guide these additional studies.

In line with this, we explored which combinations of 2 or 3 antigens gave 100% protection. We observed 913 combinations of 2 or 3 responses associated with 100% protective efficacy. To more carefully interpret the data, we examined the frequency of any antigen being randomly included in any of these protective combinations. Responses to PF3D7_0525800(IMC1G) (45%), MSP5 (27%), MSP7 (19%), MSP11 (18%) and ISP3 (11%) occurred more frequently than by chance. In our opinion this strengthens the selection of these antigens as important for future vaccine development.

To fully respond to the reviewer's concerns, we also agree it would be helpful to include a supplementary figure with a 33 x 33 matrix highlighting which combinations of 2 antigens their respective protective efficacy.

We propose the figure below, which compliments our combinations analysis showing that antigens PF3D7_0525800(IMC1G), MSP11, MSP5, MSP7, were most frequently associated with 100% protection. Please see supplementary figure S6

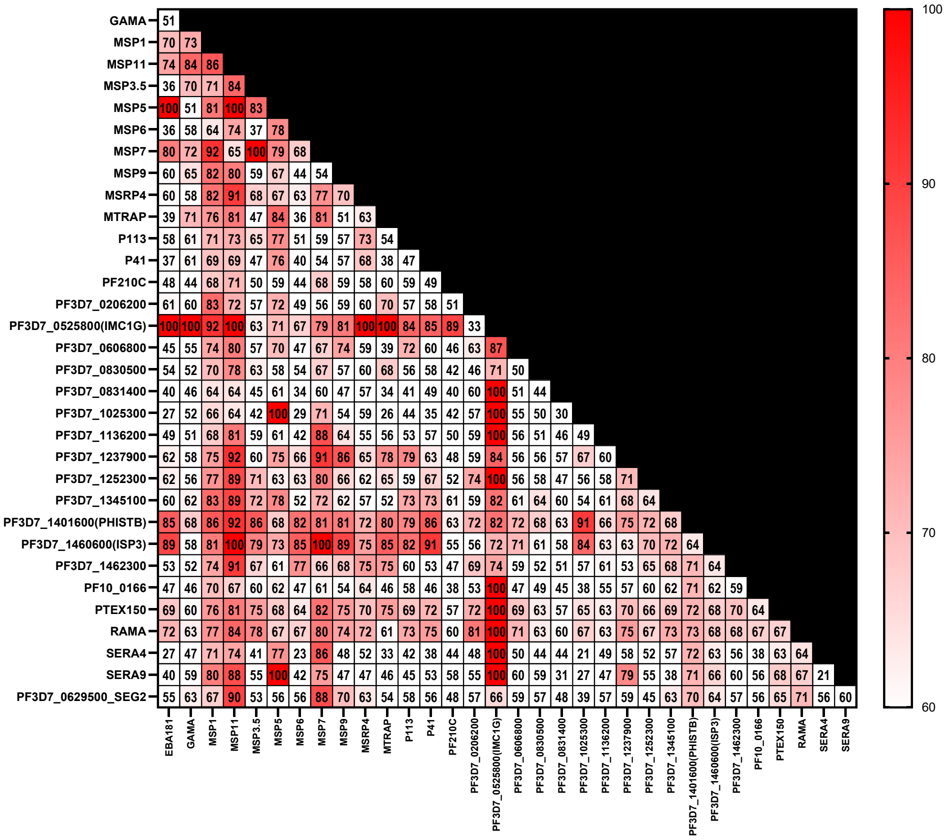

Fig S6: *Matrix showing protective efficacy ((1-RR)*100) for different antigen combinations. Boxes in red show combinations with a protective efficacy of ≥60 while all boxes in white show combinations with a protective efficacy ≤60*

1.5 Reviewer comments

While one of the strengths of this study lies in the cohort that the authors use, they do not provide much background information about this is cohort. The cohort characteristics and parasitaemia over time may have been published earlier, but it would be very helpful to provide these data here in a supplementary figure, so readers do not have to search elsewhere for this information. The parasitaemia over time, grouped by CI vs NI, would be helpful. Related to this point, were there volunteers that cleared infection and did these volunteers have different immune signatures compared to volunteers that stayed clinically immune but still had substantial parasitaemia?

Author response

We regret not providing sufficient background about the cohort and have now provided this in the methods section. We have equally included a supplementary figure S1. In line with the reviewers request, we have now included the parasite growth curves in the supplementary information.

To answer the question *related to whether volunteers that cleared infection had different immune signatures compared to volunteers that stayed clinically immune but still had substantial parasitaemia?* We plot a heat map showing the none immune subdivided into those that became febrile and those in whom parasite grew above the threshold for treatment (500parasites/µl) and clinically immune into participants that either maintained parasites below the threshold and those that were PCR negative through 21 days of follow-up. The heat map demonstrates increased breadth of responses among PCR negative individuals compared to chronic, susceptible and febrile individuals respectively. We include this as part of supplementary figure S3

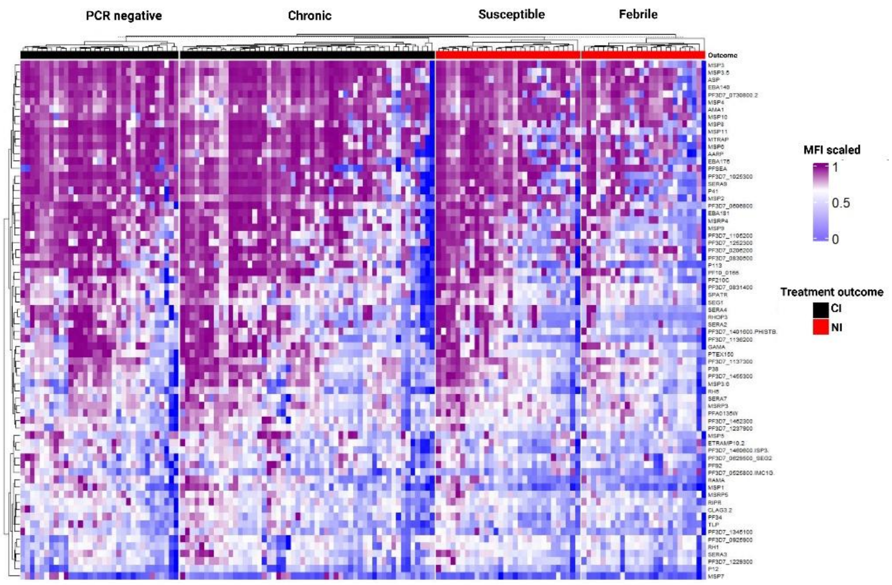

1.6 Reviewer comments

For vaccine development it is obviously important that the targets are genetically conserved, which has been a challenge in particular for blood stage antigens. Therefore it is valuable that the authors include a genetic analysis of the identified targets. However, this section of the manuscript requires substantial attention. dNS/dS values are indeed a good indication for immune pressure, but when discussing overall conservation, it is more meaningful to look at non-synonymous SNPs per coding sequence length. Also, values for NS/S SNP ratio's in table S3 do not seem to be correct (assuming that # NS SNPs and total SNPs are correct). E.g. only one quarter of the RH5 SNPS are NS SNPs (22 out of 95) so a value of 11 for NS/S SNP ratio does not seem to make sense.

Author response

We appreciate the correction and regret the errors made. We have re extracted the data from latest version of PlasmoDB and inserted a column non-synonymous SNPs per coding sequence length. Please see supplementary table S3.

Please see line 205-212: we have corrected and included the following.

*"MSP1 is under high immune pressure with a non-synonymous to synonymous single nucleotide polymorphisms ration (dN/dS) of 3.59 compared to 0.73 for PfRh5. The remaining antigens prioritized in our analysis (5/6) of those consistently identified across all methodologies and 5/5 of those occurring at a frequency of >10% in combinations) had dN/dS ratios between 0.25 (ISP3) and 2.9 (MSP7). Further their non-synonymous SNPs per coding sequence length ratios were MSP11 (0.0074), MSP7 (0.027), MSP5(0.0073), IMC1G(0.005), and ISP3 (0.0022) compared to 0.01 for PfRh5 suggesting that they are relatively conserved (**table S3**).*

Gene ID	Product Description	Gene Name	Non-Coding	NonSynony	Synonymou	NonSyn/Sy	Total SNPs	CDS Length	NonSynony	# TM Doma	SignalP Peptide	Genomic Le	P.falciparu	P.falciparu	Protein Len	Molecular Weight
PF3D7_0102500	erythrocyte binding antige	EBA181	248	66	55	1.2	369	4704	0.014031	2	MKGKMNMCLFFFYSILYVVLCTYVLG	8512	0.999	-2.102	1567	181150
PF3D7_0104200	StAR-related lipid transfe	N/A	172	25	25	1	222	1401	0.017844	0	N/A	2616	0.28	-3.379	466	53626
PF3D7_0206200	pantothenate transporter	PAT	123	4	11	0.36	138	1698	0.002356	11	N/A	2642	1	-2.212	565	62662
PF3D7_0206900	merozoite surface protein	MSP5	101	6	8	0.75	115	819	0.007326	2	N/A	1767	0.988	-2.098	272	30974
PF3D7_0207700	serine repeat antigen 4	SERA4	56	66	87	0.76	209	2889	0.022845	0	MKIHIFLIATIYVLFSEKLIKWTTA	3721	1	0.947	962	108681
PF3D7_0404900	6-cysteine protein P41	P41	155	12	6	2	174	1137	0.010554	0	MKGVIFCLVVLLWRQAWVSS	2348	0.799	-3.133	378	43088
PF3D7_0525800	inner membrane complex	IMC1g	81	5	13	0.38	99	903	0.005537	0	N/A	1895	0.244	-2.655	300	34341
PF3D7_0606800	VFT protein	VFT1	115	1	14	0.07	133	900	0.001111	0	MMHIFCKLFLFFSFVYI	2682	0.124	-2.931	299	34503
PF3D7_0629500	amino acid transporter A	AAT1	141	7	14	0.5	162	1821	0.003844	9	N/A	4212	0.143	-2.882	606	68858
PF3D7_0707300	rhoptry-associated meml	RAMA	253	77	46	1.67	379	2586	0.029776	0	MNVLLLSLLVVQNIVTYLEQ	5340	1	-1.474	861	103641
PF3D7_0828800	GPI-anchored micronema	GAMA	117	38	67	0.57	222	2217	0.01714	2	MKYYTSLYVALIIALCQAVSA	4046	0.183	-3.301	738	85250
PF3D7_0830500	sporozoite and liver stage	TryThrA	101	56	63	0.89	220	2028	0.027613	1	N/A	3863	0.223	-2.668	675	80443
PF3D7_0831400	Plasmodium exported prc	N/A	101	27	14	1.93	142	909	0.029703	1	MMRILYTNVFLFIAVLFCILSPGNNP	2341	0.969	-0.655	302	36413
PF3D7_0902800	serine repeat antigen 9	SERA9	88	27	26	1.04	141	2799	0.009646	0	MKVSFTLFFIIYIILNCDVLKC	4284	1	0.045	932	105544
PF3D7_0930300	merozoite surface protein	MSP1	96	312	87	3.59	495	5163	0.06043	1	MKIIFFLCSFLFFIINTQC	6272	0.141	-3.197	1720	195726
PF3D7_1017100	rhoptry neck protein 12	RON12	166	4	9	0.44	179	933	0.004287	1	MKRVYTCLCFICLFFLCILKGVYN	2717	0.13	-2.976	310	36405
PF3D7_1025300	conserved protein, unkno	N/A	35	23	23	1	83	2769	0.008306	0	MLLYFVIFIWSFLLCDI	3079	0.121	-3.037	922	109073
PF3D7_1028700	merozoite TRAP-like prote	MTRAP	180	36	25	1.44	241	1497	0.024048	1	MKKTILNLYLINILFALSDVKG	3913	1	-1.824	498	58085
PF3D7_1035500	merozoite surface protein	MSP6	48	32	25	1.28	105	1116	0.028674	0	N/A	2396	0.565	-2.477	371	42275
PF3D7_1035800	probable protein, unknow	M712	40	73	136	0.54	249	2139	0.034128	0	MKYILSISLFLILLNLYKC	3588	1	-2.143	712	82071
PF3D7_1036000	merozoite surface protein	MSP11	62	9	4	2.25	75	1218	0.007389	0	MNKFLNIIFYIFLILNFSFFQSNA	2490	0.391	-2.514	405	46729
PF3D7_1136200	asparagine-rich merozoit	ARMA	81	44	46	0.96	171	2040	0.021569	0	N/A	3154	1	-2.026	679	76570
PF3D7_1228600	merozoite surface protein	MSP9	27	41	50	0.82	118	2232	0.018369	0	MMNMKIVLFSLLLFVIRWNIISC	2611	0.39	-3.212	743	86623
PF3D7_1237900	conserved Plasmodium p	N/A	61	132	179	0.74	372	3567	0.037006	1	N/A	4117	1	-0.82	1188	140939
PF3D7_1252300	conserved Plasmodium p	N/A	153	2	0	0	155	258	0.007752	1	N/A	3141	1	-1.546	85	9443
PF3D7_1334400	MSP7-like protein	MSRP4	0	17	4	4.25	21	930	0.01828	0	MKGRIISFSFFLFCMVHFVFC	930	0.12	-3.036	309	36383
PF3D7_1335100	merozoite surface protein	MSP7	111	29	10	2.9	150	1056	0.027462	0	MKSNIIFYFSFFFVYLYYVSC	3340	0.459	-2.577	351	41276
PF3D7_1345100	thioredoxin 2	TRX2	46	4	4	1	54	474	0.008439	0	MKKYIFFFLFSFINFFFVYDVTC	1223	0.122	-3.166	157	18630
PF3D7_1401600	Plasmodium exported prc	N/A	187	19	13	1.46	219	1437	0.013222	1	N/A	4392	1	-1.412	478	55827
PF3D7_1420700	surface protein P113	P113	153	32	37	0.86	222	2910	0.010997	1	MKIPFFILHILLLQFLLCLIRC	6012	0.991	0.436	969	112574
PF3D7_1436300	translocon component PT	PTEX150	211	36	26	1.38	273	2982	0.012072	0	MRIIILALLIVCTIINYYCA	7350	1	-2.182	993	112409
PF3D7_1460600	inner membrane complex	ISP3	86	1	4	0.25	92	447	0.002237	0	N/A	1679	0.567	-2.264	148	17117
PF3D7_1462300	GTP-binding protein, puta	N/A	55	27	62	0.44	144	4074	0.006627	3	MNKLFFLCFLVIIIYSLS	5486	0.178	-3.307	1357	161219
PF3D7_0424100	reticulocyte binding prote	RH5	57	16	22	0.73	95	1581	0.01012	0	N/A	2460	0.126	-3.141	526	62996

Other points:

1.8 Reviewer comments

- Figure 2A – There seem to be 71 antigen reactivity rows, rather than 70.

Thank you for pointing this out the figure has been corrected. Please see revised figure 2A

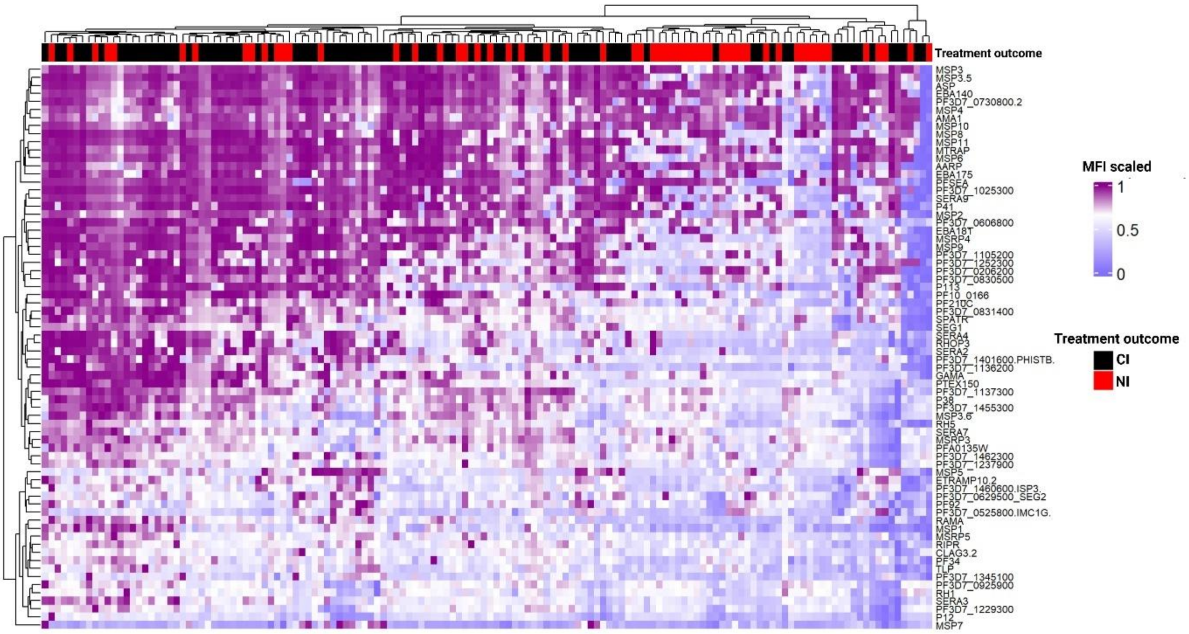

1.9 Reviewer comments

- Figure 2A – Text in top right corner "groups" is not aligned properly

These have been corrected. Please see the revised figure above.

1.10 Reviewer comments

- Figure 2A – Could a supplementary figure be added that shows the same data, but with the immune and non-immune people grouped? This would be helpful to compare responses within groups

We have included a figure with clinically immune and non-immune people grouped together (reproduced below). Please see supplementary figure S3

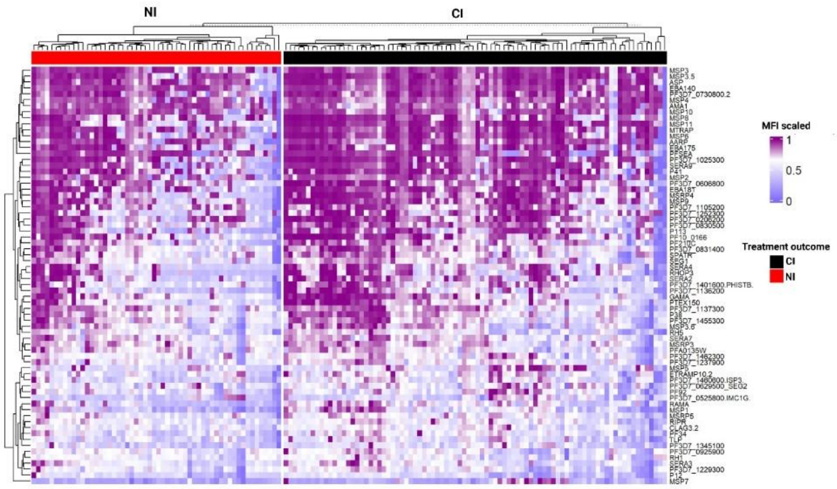

1.11 Reviewer comments

- Legend Figure 2A – “Treatment outcomes are annotated in the two rows”, there seems to be only one row.

This has been corrected

1.12 Reviewer comments

- Figure 3A X axis should be corrected from Not treated-Treated to Clinical Immune- Non Immune.

This has been corrected, Please see corrected figure 3A

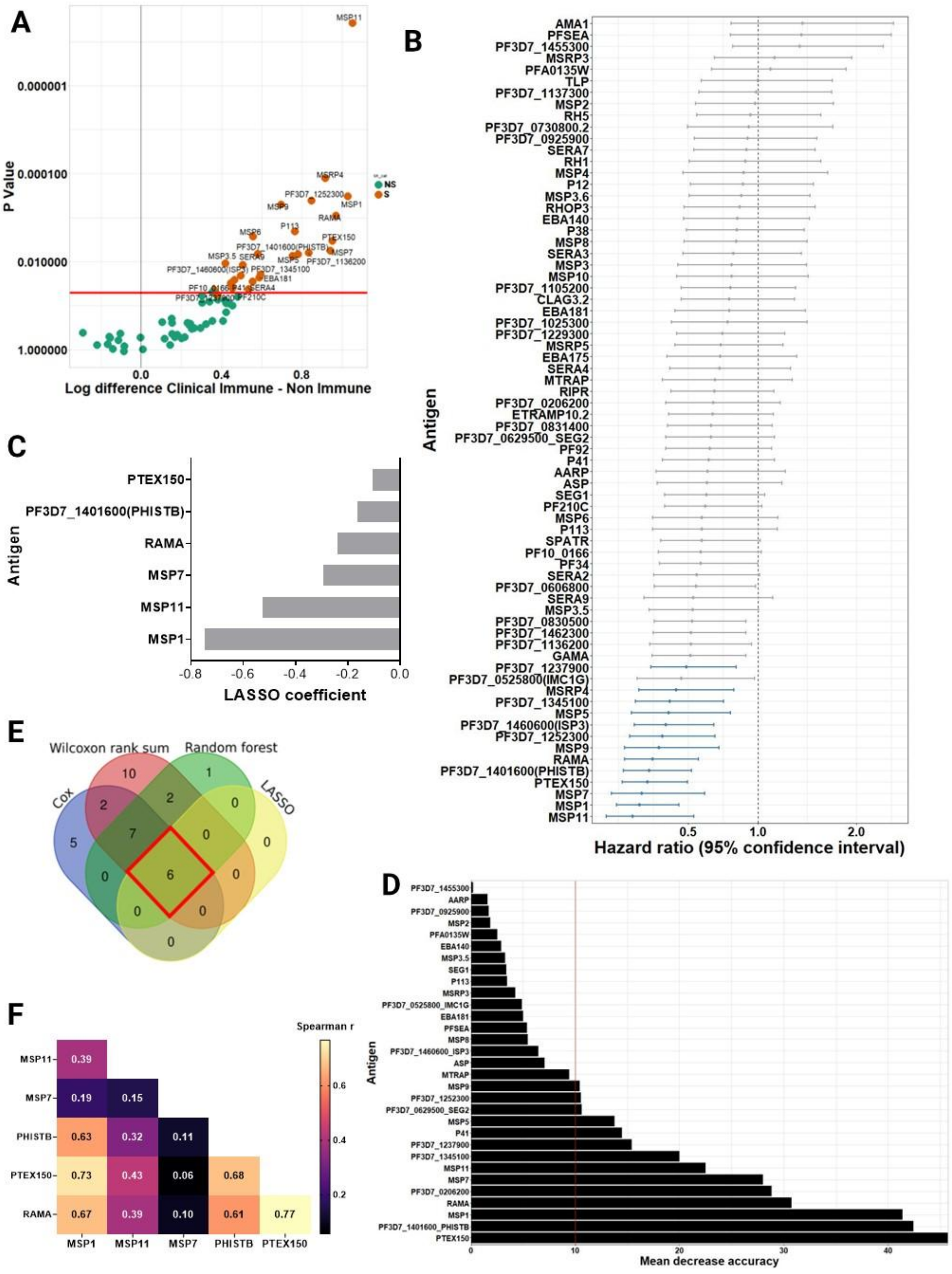

1.13 Reviewer comments

- Figure 4B is related to Figure 3, and Figure 4A and 4C are related. Therefore, it seems sensible to change the order of the figures, either by swapping 4A and 4B or integrating 4B in Figure 3.

Thank you for this helpful suggestion. We have swapped the order of the figures. Please see this below.

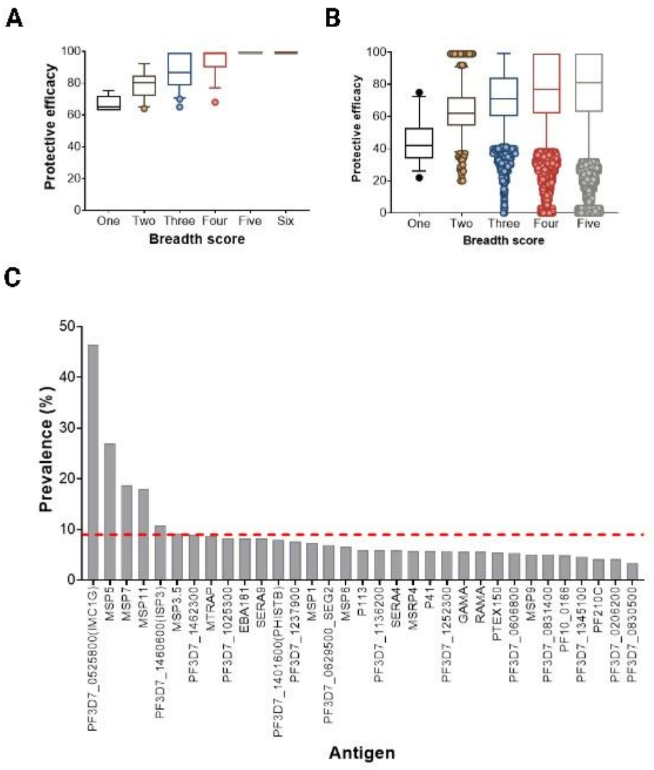

We equally have revised the corresponding text within the manuscript. Please see page 9 line 181 -191

*We first tested combinations of the six antigens consistently ranked at the top (fig 3E) and thereafter explored the 33 antigens identified as important across all analytical methodologies. For both analyses, protective efficacy (defined as (1 – RR) %) increased with breadth, defined as the sum of individual antibody responses. In the first analysis with only the top six antigens, responses to a combination of four antigens was associated with a median protective efficacy of 100% **(fig. 4A)**. In the second analysis including 33 antigens, rare combinations of responses to just two or three antigens were associated with up to 100% protective efficacy **(fig. 4B)**.*

1.14 Reviewer comments

- Legend Figure 4A: comparison "between individuals with high versus low-to-moderate antibodies". Define high and low-to-moderate cutoff.

Included

1.15 Reviewer comments

- Legend Figure 4A: correct typo in "Combination analysis the top 33 candidates"

We regret this mistake. This has been corrected it now reads

"Combination analysis for the top 33 antigens identified as important across all analytical methodologies"

1.16 Reviewer comments

- Specify which version of PlasmoDB has been used for retrieving sequence data to do the sequence analysis.

Thank you for this correction. We reextracted this data using PlasmoDB release 68 (7 may 2024). This has been included within the manuscript please see page 9 line 187

1.17 Reviewer comments

- Lines 199-200: names of top 6 antigens changed to MSP1, MSP11, MSP5, IMC1G and ISP3, whereas everywhere else they are MSP1, MSP11, RAMA, PTEX150 and PHISTb.

Thank you for highlighting the inconsistency. We have corrected this

Please see page 9 line 217 – 222. We have corrected to read as follows:
*Likewise, the mutant fitness score (MFS) was <-2, indicating a growth fitness cost for mutating the genes. For the top six antigens identified using multiple analytical strategies (MSP1, MSP11, RAMA, MSP7, PF3D7_1401600 (PHISTB) and PTEX150,) as well the five antigens identified through our combinational analyses (MSP11, MSP7 MSP5, IMC1G and ISP3)   the MIS ranged from a low of 0.14 for MSP1 to 1.0 for RAMA, PTEX150, while MFS ranged from -3.19 – MSP1 to -1.47 -RAMA (**table S3**). Both the mutagenesis index and the mutant fitness scores were comparable to PfRh5 (MIS = 0.126 and MFS = -3.14) (29).*

1.18 Reviewer comments

- It is not clear how many antigens have been excluded from analysis. Line 88 says 70 antigens out of 110 were included for analysis, but methods only refer to 25 being excluded, making a total of 85 antigens to include for analysis.

We regret this error. We included 70 antigens within this analysis. We have corrected this to read 70 of 95. Please page 5 line 89

Reviewer #2 (Remarks to the Author):
Introduction
The authors previously conducted controlled human malaria infection (CHMI) studies in Kenyan adults and demonstrated that clinical immunity was strongly correlated with IgG Fc-mediated functional activity against whole merozoites. In this study, they utilized a KILchip system to identify the merozoite antigens that induce antibodies associated with clinical immunity observed in the CHMI study. Using five distinct methodologies, they consistently identified six key antigens: MSP1, MSP11, RAMA, MSP7, PF3D7_1401600, and PTEX150. Selected combinations of these antigens predicted 100% protection against malaria. Although all the studies are carefully designed and conducted, the primary objective—an examination of the IgG Fc-mediated functional response—was not directly addressed. Additionally, the

contribution of the selected antigens to the IgG Fc-mediated functional response has not been experimentally validated. I have comments to improve this manuscript.

Author response

We agree with the reviewer's assessment. This was equally raised by reviewer one. As detailed above in response to points 1.2 AND 1.3, we have revised the manuscript removing mention of the aim to identify combinations of antigens that are associated with protection, with the aim of informing further pre-clinical and clinical development. We hope that this rewording more clearly describes the content of the manuscript. Please see reviewer comment 1.2

Major Comments:

2.1 Reviewer comments

1) Lines 66-68: The authors stated the objective of their study: "We therefore sought to identify additional merozoite antigens that, in combination, would potentially increase the potency of the IgG Fc-mediated functional response." However, they only measured the levels of IgG against 70 Pf antigens using the KILchip system. To meet their objectives, they should assess the Fc-mediated functional response for at least one of the selected antigens beyond MSP1, as they are the leading experts in this research field. This step is crucial for validating their selection results.

Author response

We appreciate this comment and agree that the objective of the paper was not accurately described as demonstrating Fc-mediated functional responses. We have revised the manuscript to represent the data presented.

Please see page 3, line 66 – 69, we have deleted the sentence "*We therefore sought to identify additional merozoite antigens, that in combination would potentially increase the potency of the IgG Fc-mediated functional response*." We have replaced this with "*We therefore sought to identify merozoite antigens, that may independently and or in combination be associated with protection to clinical malaria* with the aim of informing further pre-clinical and clinical development*"*

In addition, we discus as a limitation and next steps the need for data to demonstrate functional capacity of the identified antigens. Demonstrating antibody functionality of our findings would be a major undertaking. One could propose mouse immunizations, or antibody purification, and then in vitro functional assays, but we do not believe there is a single canonical functional assay that could be used as a definitive answer, and we would need to consider a number of antigens and potentially combinations (see below). This will require substantial further funding and development. We would suggest that taken together, the identification of novel antigen targets with apparent associations with protection, and the identification of potentially synergistic combinations of antigens, is relevant to the malaria vaccine community and will stimulate further lines of research with differing vaccine platforms and experimental approaches.

2.2 Reviewer comments

2) One of the top six antigens, PTEX150, was selected. However, this protein is well-known as one of the components of PTEX, which is involved in the trafficking of the parasite's proteins to the red blood cell (RBC) cytoplasm. There is no interpretation or discussion regarding the significance of selecting PTEX150 in this study.

Author response

We regret not including a discussion on PTEX150. Please see page 13 line 281-287. We have include the following.

PTEX150 is part of the Plasmodium translocon of exported proteins (PTEX) complex on the parasitophorous vacuole membrane and plays a crucial role in protein export to the infected red blood cell membrane(38, 39). More recently it has been shown to also be essential for parasite growth within hepatocytes (40). At the merozoite stage, PTEX150 is localized within dense granules alongside other members of the PTEX complex which are later released into the parasitophorous vacuole (41). However, the secretion or localization of PTEX150 on the merozoite surface during the invasion process is unknown.

2.3 Reviewer comments

3) In this study, "protection" refers to the "protection against IV PfSPZ challenge." Therefore, I believe that including PfCSP as one of the test antigens is essential.

Author response

The chip was previously designed to investigate immunogenic merozoite surface antigens. We agree that PfCSP responses will be interesting, but they do not complicate our analysis here given our challenge route and their levels in the population, and would require a different experiment. We have recently shown (Kapulu, M.C., Orenge, F., Kimani, D. et al. Malaria vaccine protection against intradermal or venous parasites: a randomized phase 2b human challenge trial. Nat Med 32, 178–185 (2026). https://doi.org/10.1038/s41591-025-04107-6) that CS antibodies are protective against intradermal but not intravenous sporozoites, and in the study we report here, all sporozoites were administered intravenously. Furthermore, the levels of CS antibodies in the unvaccinated population are several orders of magnitude below those induced by the R21 vaccine, and hence there is little formal prospect of these antibodies being protective in the present experiment.

Please see page 14 line 325 -327.

We insert the following. "The microarray was designed to investigate merozoite antigens however as the challenge was IV PfSPZ further studies investigating sporozoite specific antigens may be required to improve our understanding. "

2.4 Reviewer comments

4) To assess the quality of proteins expressed in the KILchip, please include the "expression

system" in Table S1 and incorporate this into the analyses. Furthermore, for proteins expressed in Expi293, does their "codon optimization" eliminate N-glycosylation?
Author response

We appreciate this comment and have included the expression system for each protein into the table S1. The design and validation of the chip has been previously published (Kamuyu G, et al.,2018). We reference this manuscript which gives a full description of the protein microarray chips design and development.

Regarding codon optimization eliminating N-glycosylation. Unlike the majority of eukaryotic extracellular proteins, *Plasmodium* cell surface and secreted proteins are not modified by *N*-linked glycans. This is because the parasite lacks the necessary enzymes (Itzstein MV et al., 2008). Therefore, during codon optimisation, we included an additional step. We substituted all serine or threonine amino acid residues in all potential N-linked glycosylation sites (NXS/T) with alanine in order to avoid unwanted glycosylation. We have made this clearer within the text.

Please page 16 line 379-381: we insert "*For mammalian expression, all sequences were codon-optimized and all potential N-linked glycosylation sites (NXS/T) modified by substituting serine or threonine residues with an alanine residue.*"

Minor comments:
2.5 Reviewer comments

Line 40: "drug-resistant parasite" should be mentioned.

Author response

Thank you for this suggestion, this has been included. Please see page 2 line 41.

The sentence now reads

This stagnation of progress has been attributed in part to the rise of pyrethroid resistance in insecticide treated nets (ITNs), drug resistant parasites, as well as health and humanitarian emergencies such as famine, flooding and disease outbreaks

2.6 Reviewer comments

Line 299: Are there any parasitic individuals at the recruitment? If so, exclude or treat them before the inclusion.
Author response
No, none was parasitaemic at recruitment. Careful screening and selection was conducted prior to recruitment and challenge. This has been made clearer with the methods section.

Please see page 16, line 354 -356 we include the following.

The study was open, non-blinded, and non-randomized. The participants, all Kenyan and of similar ethnicity, were carefully screened and recruited from different areas of the country to provide varied previous malaria exposure. Those with sickle cell trait, known to influence malaria infections, and active asexual parasites were excluded

Reviewer #3 (Remarks to the Author):

The study leverages a large human challenge model in semi-immune population to identify IgG responses that account for natural acquired immunity against Plasmodium falciparum clinical episodes. Researchers utilized a custom merozoite protein microarray (KILchip) to screen immune responses in semi-immune Kenyan adults and employed five complementary statistical methods—including traditional models and machine learning approaches—to prioritize antigens associated with clinical immunity. Six antigens (MSP1, MSP11, RAMA, MSP7, PF3D7_1401600 (PHISTB), and PTEX150) were consistently associated with protection across all analyses. Moreover, the use of combinations of antibody responses was more predictive than a single antigen, suggesting that independent contributions to protection may be at play. The study identified antibodies against Pf merozoite antigens that had been both reported as markers of naturally acquired immunity in previous studies and others that had not yet been reported. Unlike observational longitudinal studies of cohorts where exposure is not controlled, results from large CHMI like this present less sources of confounding and are of great value. These findings are important to identify new vaccine candidates

However, this study presents important shortcomings in the reporting and in the methodology:

We thank the reviewer for the positive review of our work. We hope the revisions made throughout the manuscript will improve the quality and message of the manuscript.

Major:

3.1 Reviewer comments

1. We miss a figure reporting the original MFI data –after pre-processing, i.e. exclusions and noise subtraction, that feed all subsequent analyses. Original data should be shown to readers in the manuscript, not in the supplementary. Furthermore, such figure would benefit from plotting separately MFI measurements from NI and CI (by colouring or plotting them as separate paired boxplots). This plot partly already exists but is placed in the supplementary (S1).

Author response

We appreciate this suggestion and regret not including this figure. We now include figure 1A showing the original MFI values separated by NI and CI.

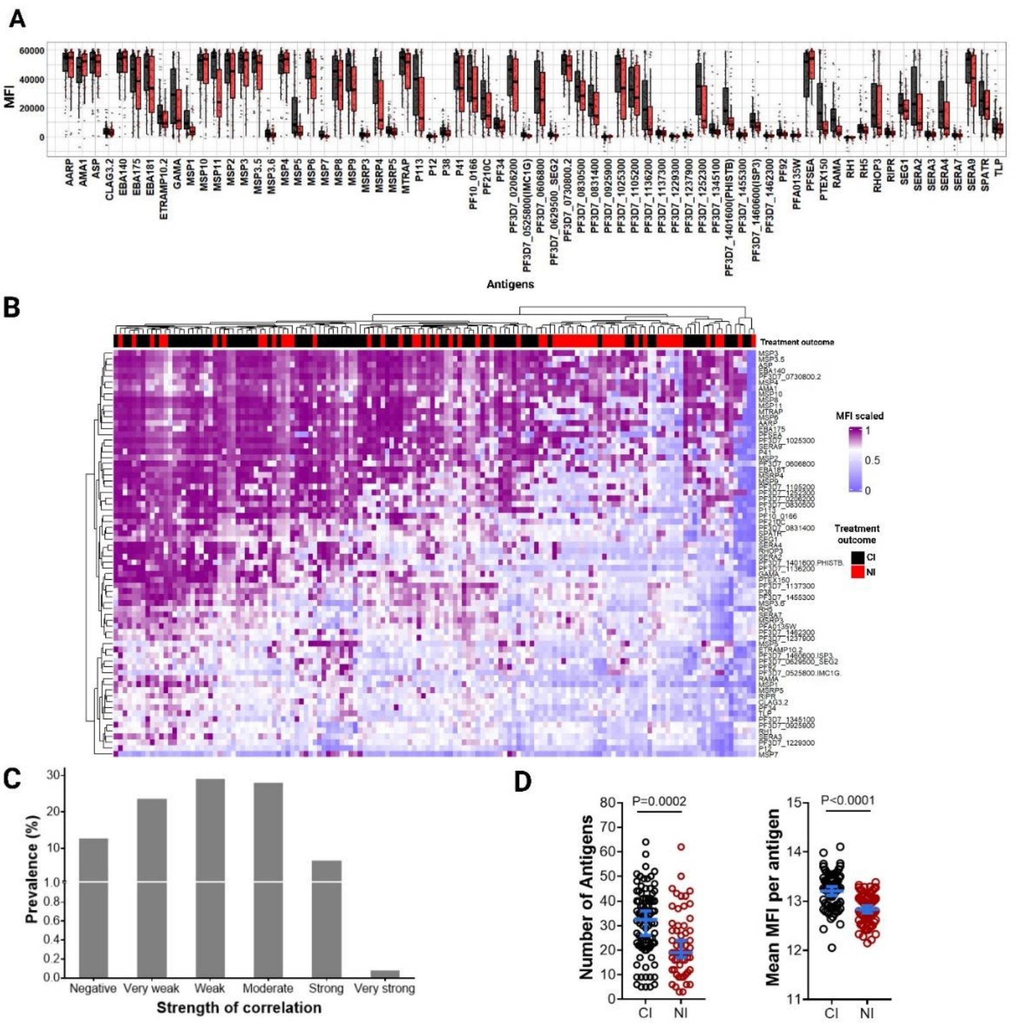

We also included within the supplementary a figure showing Log transformed and variance stabilization normalization (VSN) corrected data.

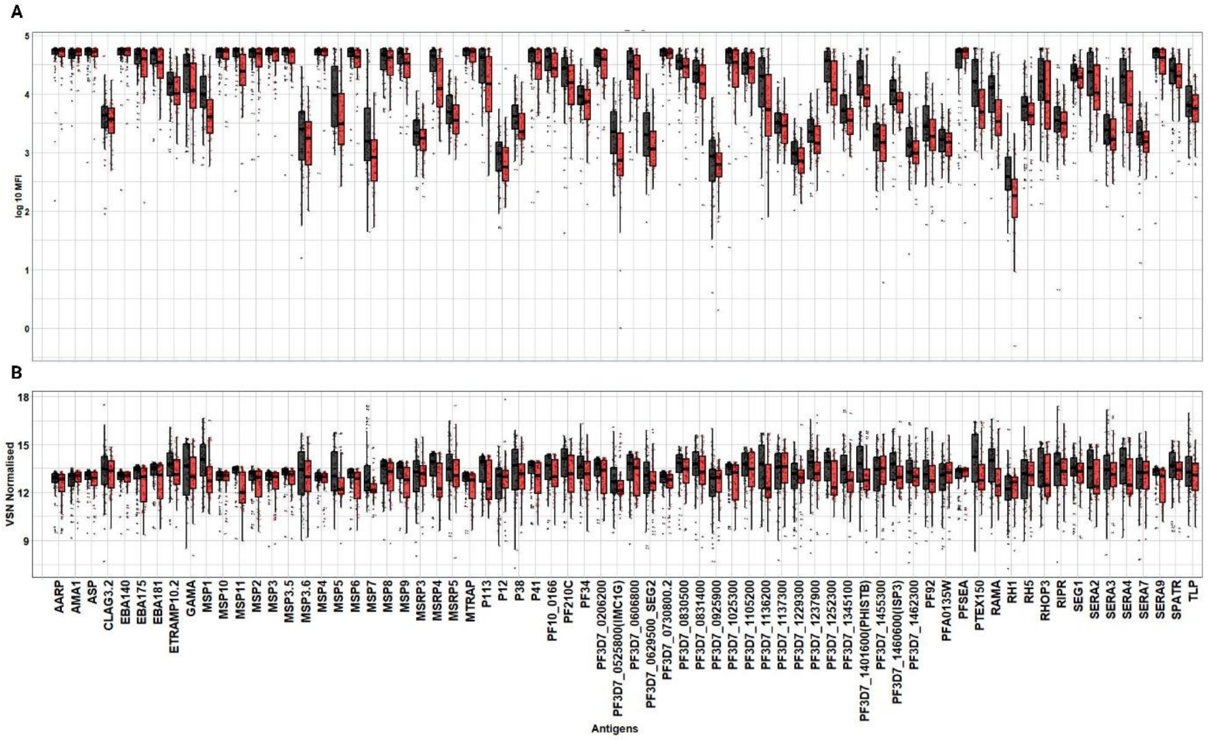

3.2 Reviewer comments

This Figure S1 raises the following issues:

1.1. There are several well-known markers of exposure (and candidate determinants of protection) that clearly have a saturated signal: AMA1, EBA140, EBA180, MSP2, MSP3, MSP4… This should be acknowledged at least as a limitation in the discussion. In Lines 113 and elsewhere I believe, the authors highlighted they did not find any association with protection for AMA1 or MSP3 as probably expected. Maybe here they have one possible explanation for this. Potential good predictors of immunity contained in the microarray may have been detected as indeed associated with protection, had they been measured in an assay or at a dilution that was able to resolve differences for higher Ab concentrations.

Author response

We agree that this may be a concern. We thus acknowledged this in the discussion. Please see page 15 line 332-335 we insert the following

"*A further possibility for insensitivity to markers of protection is saturation of signal. This is suggested for the following antigens (AMA1, EBA140, EBA175, EBA180, MSP2, MSP3, MSP4,), where distributions were truncated at the upper limits of the assays dynamic range.*

We do not see the manuscript as ruling out well-known antigens given the limitations described here and elsewhere in the manuscript, but rather as suggesting additional antigens for consideration that may not yet have been included in pre-clinical development.

3.3 Reviewer comments

1.2. As expected, Figure S1 shows that the MFI measurements follow a rather log-normal distribution, and thereby log-transformation of MFI measurements seems well-justified (which incidentally was done in the Figure S1). However, in most analyses in the manuscript, the authors preferred to use natural scale MFI values. For antibody levels/concentrations, researchers usually compare geometric means instead of arithmetic means and use log-transformed values as covariates in their (generalized) linear models. The authors should make clearer in the methods that they indeed do not use log-transformed MFI values and should provide a convincing rationale for this unusual choice.

Author response

We appreciate the opportunity to clarify. Our analyses uses data obtained after conducting a variance stabilization normalization method (VSN).

We previously explored four (VSN,Log2, RLM and cyclic loess) functions for normalization to identify one that optimally reduced the mean–variance dependence (MVD) (Mwai k., et al 2021). We formally assessed this using mean versus standard deviation plots (*meanSdPlots*) and coupled this to automatically derived Spearman correlation estimates (*Rho*) and Cox–Stuart or the Mann–Kendall trend tests. The latter quantifies the performance of the normalization. Implementation of the $\log_2$ approach led to an inflation of variance for the low MFI (Mean Fluorescent Intensity) values. The cyclic loess and RLM were not optimal. Consequently, we adopted the VSN normalization approach. The VSN method overcomes the limitations of log transformations minimizing the inflated variance around low signal intensities, but also results in normalization as effectively as log-transformation. It calibrates between-feature variation through shifting and scaling mechanism in which all the data are adjusted. Huber et al. and Durbin et al. independently proposed the VSN approach for such data which is a variant of the log-transform (*glog2*).

Please see page 5, line 92-101 where we clarify this by inserting the following

"The level of antibody reactivity against each antigen varied; some antigens showed a high level of reactivity and were present in a high proportion of volunteers, while responses to others were less strong and detected in fewer volunteers (fig 2A). We previously explored four (variance stabilization normalization (VSN), Log_2, robust-linear-model (RLM) and cyclic loess) functions for normalization to identify one that optimally reduced the mean–variance dependence (MVD) (23). The cyclic loess and RLM were not optimal. Consequently, we adopted the VSN normalization approach that overcomes the limitations of log transformations minimizing the inflated variance around low signal intensities, but also results in normalization as effectively as log-transformation. Hereafter all analyses were conducted using VSN normalized data (fig S2).

Please see page 19 line 439- 451. We insert this expanded explanation within the statistical analyses section

"We previously explored four (VSN,Log2, RLM and cyclic loess) functions for normalization to identify one that optimally reduced the mean–variance dependence (MVD) (23)*. We formally assessed this using mean versus standard deviation plots (meanSdPlots) and coupled this to automatically derived Spearman correlation estimates (Rho) and Cox–Stuart or the Mann–Kendall trend tests. The latter quantifies the performance of the normalization. Implementation of the log_2 approach led to an inflation of variance for the low MFI (Mean Fluorescent Intensity) values. The cyclic loess and RLM were not optimal. Consequently, we adopted the VSN normalization approach. The VSN method overcomes the limitations of log transformations minimizing the inflated variance around low signal intensities, but also results in normalization as effectively as log-transformation. It calibrates between-feature variation through shifting and scaling mechanism in which all the data are adjusted. Huber et al. and Durbin et al. independently proposed the VSN approach for such data which is a variant of the log-transform (glog2)..."*

3.4 Reviewer comments

The method to assess the accuracy for predicting clinical immunity using a combination of Ab responses is complicated, not well described and may potentially be too optimistic because it may suffer from overfitting, as the authors do not validate or cross-validate the whole process.

Author response

We agree that our analysis may suffer from overfitting, and that we lacked a validation cohort. We now acknowledge this as a limitation.

Please see page 15 line 335-339. We include the following sentence

In addition, our analyses may suffer from over-fitting, misclassification biases or over-simplification. Especially as regards the combination analysis where multiple antigens are possible. We do not suggest that any combination are likely to be associated with 100% protection, rather we are using this analysis to highlight combinations of antigens that merit further investigation.

3.5 Reviewer comments

Lines 413, how are high and low Ab levels determined? I cannot see it described in the methods or elsewhere. Is this determined based on the linear combination of log (MFI) of the different Ab using the coefficients obtained from the modified Poisson regression applied repeatedly on all the studied combinations? If so, what is the threshold for classifying between high and low using this linear combination?

Author response

We determine High vs low antibody level by ranking responses for each antigen and then dividing the data into tertiles. The individuals in the top tertiles were then assigned as high and those in tertile 2 and 3 low.

Please see page 21 line 464-466 we included

High vs low to moderate antibody level were determined by ranking responses for each antigen and then dividing the data into tertiles. The individuals in the top tertiles were then assigned as high and those in tertile 2 and 3 low to moderate.

3.6 Reviewer comments

I do not understand the purpose of fitting models using an increasing number of Abs in one case from the final subset of best predictors and from the total 33 Abs and then compare one procedure with the other. Of course, average prediction scores will be better when using combinations from the subset of best predictors. This is a trivial result

Author response

We agree it is a statistical inevitability that combinations will predict higher levels of protection. We did not intend to test the hypothesis that combinations would increase protection compared with single antigens, but rather to select those antigen combinations that seemed to result in the highest increases in protection. We do not consider these findings conclusive but more a means to suggest further lines of investigation.

Please see page 13 line 301 -311

Given the complexities of vaccine development, the antigens that we have identified as having potential for combination or multivalent vaccines will need further reduction and refinement. We consider these data as important first steps. Further epitope mapping and structural level characterization will need to be applied to elucidate and subsequently validate the critical epitopes necessary for protection and amenable to vaccine development. Further studies would include vaccination of mice to determine functional immunity and epitope and structural level characterization of these antigens may elucidate the critical epitopes required to mediate protection. Antigens raising non-functional responses would be de-prioritized, and further challenge studies with multiple combinations would identify the minimum number of antigens required for further clinical development. We would prioritize testing combinations of 2 or 3 antigens that were associated with the highest levels of protection in our study.

3.7 Reviewer comments

The formula for obtaining the "protective efficacy" is (1-RR)*100. The authors should clarify where the RR estimates come from. Do they come from the exponentiated modified Poisson regression coefficients of a binary covariate (high/low antibody responder given a combination of Ags?). Please clarify? Be as it may, you should provide confidence intervals for your RR estimates.

Author response

Yes, they are from exponentiated modified Poisson regression coefficients of a binary covariate (high/low antibody responder given a combination of Ags) High vs low to moderate antibody level were determined by ranking responses for each antigen and then dividing the data into tertiles. The individuals in the top tertiles were then assigned as high and the rest as low. Please see comment 3.5 above and page 21 line 464 – 470.

All confidence intervals are available in figures and in tables.

3.8 Reviewer comments

2.4. More specifically, reporting RR (or, what is equivalent, efficacy as 1-RR) in this context can be misleading, especially if we do not know which individuals are classified as high and low responders. You may have a small group of so-called high responders that are all CI, whilst the low responders contain both NI and CI. RR of high over low responders will then be 0, yet both the true positive rate and the true negative rate of your Ab-based prediction may be considerably lower than 100%. This may explain the surprising result that whereas you can achieve 100% efficacy with only 2 Ag, the LASSO penalized regression chose 6 regressors. This is because 100% efficacy does not mean 100% accuracy, and the departure from one another can be very large.

Author response

The high vs low classification is based on tertiles (please see comment 3.7 above) and so the scenario of a small group of high responders may not arise. Furthermore, our prior analyses identified protective signals for specific antigens using the response as a continuous variable, without the division into tertiles, hence it is unlikely that our findings are driven by an artefact of the distribution of responses.

However, we do agree that the finding of 100% protection with a few antigen combinations seems surprising, and we suggest that this may be due to overfitting and multiple comparisons with multiple antigen combinations. Please see page 15, line 321-329, we acknowledge this limitation as follows:

In addition, our analyses may suffer from over-fitting, misclassification biases or over-simplification, especially as regards the combination analysis where multiple antigen combinations are possible. We do not suggest that any combinations are likely to be associated with 100% protection, rather we are using this analysis to highlight combinations of antigens that merit further investigation.

3.9 Reviewer comments

In studies assessing the prognostic (or diagnostic) predictive power of biomarkers, one would

expect to report the true positive rate (sensitivity) and true negative rates (specificity) of your multi-Ag panel to predict clinical immunity. Providing ROC curves would have been nice, by sliding the threshold separating high and low Ab groups. Additionally, whatever prediction score you choose to report, it should be validated with a validation subset unseen by the algorithm (including the filtering process that the authors used to select the final 6 best predicting Ags) or using cross-validation. In such studies, the greatest danger is always overfitting.

Author response

We certainly agree with the reviewer that overfitting maybe an issue and acknowledge this within the discussion. Please see page 15 line 335. *". In addition, our analyses may suffer from over-fitting, misclassification biases or over-simplification."*

However, to reduce the bias of overfitting we used a regularization model (LASSO) and conducted a 10-fold cross validation. Please see page 20 line 448-452 where we mention

"The random forest model was optimized using a 10-fold cross-validation loop to get the lowest out of bag error and highest accuracy. The best model had 10,000 trees (mtree) with 48 random antigens per tree (mtry). The average error for the forest was analysed, and the importance for each variable was determined by comparing the out-of-bag error before and after permutation over all trees and a score assigned. The score was normalized over the standard deviation, and the variable with the highest score was ranked as the most important."

3.10 Reviewer comments

3. Lines 385-391: Why do authors adjust the p-value for multiple comparisons for method 1) but not for method 2) and 3), unless they do, and they didn't specify it in the manuscript (Figure 3A-C)? Modified Poisson and PH Cox regressions are conducted repeatedly for each Ag, therefore they should also be adjusted for multiple comparisons to report findings with a controlled false discovery rate.

Author response

We regret this omission as we adjust for multiple comparison in all analyses. We have corrected this throughout the manuscript.

3.11 Reviewer comments

4. The study places a lot of emphasis on the use of "independent analytical approaches", implying that converging results will necessarily be robust and method-independent. But the statistical and machine learning methods they used are very different to each other and respond to different objectives. They are necessarily bound to yield different results. These results are not necessarily incorrect because they do not agree with each other. They simply respond to different questions. The authors themselves acknowledge this : "…in focusing on the six antigens that were consistently identified, it is likely that we are excluding antigens that were genuinely associated with outcome but where methodological issues led to

erroneous exclusion in one analytical approach". It is not likely, the chosen strategy will necessarily do this.

For instance, whilst the univariate methods: 1, 2 and 3 are intended to detect associations with protection with a given false discovery rate, and, to a certain extent, they should be quite comparable provided p_values are corrected for multiple comparisons in all of them, methods 4 and 5 are of a completely different nature. They simply maximize prediction and will select whichever Ags are best (and not redundant) at achieving it. Furthermore, the lasso-penalised regression will also try to achieve this with as few Ags as possible. Therefore, if a lasso-penalised regression does not select a certain number of Ags, this is by no means evidence that the excluded Ags are not associated with protection. Simply, it means that the algorithm could predict immunity by means of other (probably redundant) responses against other Ags. I can see numerous Ab responses against Ags that are significantly and quite strongly associated with protection using methods 1,2 and 3, but are excluded from the final 6-Ag set for this reason. Is this not giving the misleading idea that they are worse vaccine candidates?

Author response

We agree that some protective antigens will have been "missed". We frame our approach as a conservative study to "ruling in" rather than "ruling out" in part because the data do not allow for a further validation cohort in which we could test less significant signals, and secondly because our objective was to identify priorities for new candidate antigens, not necessarily to rule out existing candidates.

Please see page 15 line 318 we include the sentence

Our approach has arguably maximized specificity over sensitivity: i.e. we have more confidence "ruling in" antigens than "ruling out".

Minor:

3.12 Reviewer comments

1. Line 91: "breadth" is defined as the number of antibody "high responses". Where in the manuscript is a high/moderate/low response defined and a method/threshold to identify them established?

Author response

We determine High vs low antibody level by ranking individual responses for each antigen and then dividing the data into tertiles. The individuals in the top tertiles were then assigned as high and those in tertile 2 and 3 low.

Please see comment 3.7 above

3.13 Reviewer comments

2. Line 99: "magnitude" is defined as the sum of MFIs. It seems you are computing the

arithmetic mean, not the geometric mean. This has to do with the fact that you usually use natural scale MFI, not log-transformed MFI. Given the distribution of MFI (see S1), the arithmetic mean will be very much (perhaps too much) driven by the far end of the upper tale.

Author response

We computed the mean of VSN normalised MFI values for all antigens. This has been made clearer within the manuscript please comment 3.3 above

3.14 Reviewer comments
3. 138-140: The description of what LASSO penalty achieves in a regression is incorrect. LASSO does not minimize variance between outcome groups as stated. Lasso tries to minimize prediction error while keeping the model simple in terms of number of predictors (sparse)

Author response

We have now corrected this within the manuscript. Please see page 7 line 141. The sentence now reads as

This penalized regression method minimizes prediction error while keeping the model simple in terms of number of predictors between the two outcome groups, NI and CI.

3.15 Reviewer comments
4. Line 142: regarding cross-validation, you said "sampling with replacement of 15 variables at a time". Do you mean 15 individuals at a time?

Author response

We refer to 15 variables – which are the different antigen responses

3.16 Reviewer comments
5. In line 377, authors state that "duplicate spots with a coefficient of variation greater than 20%" were re-assayed. Which duplicates are the authors referring to? Blank controls, protein tag controls or all test samples that were measured in duplicates? Authors should explain this.

Author response

We regret that this was not clear. We have clarified this by first. Stating that all proteins were printed in duplicate spots. Please see page 18 line 389

Proteins were printed in duplicate at a concentration of 250 µg/ml using the Ultra Marathon micro-arrayer (ArrayJet) with the Inkjet printing technology and the command centre 1.5.0.1 (ArrayJet)

We then clarify that signal level from duplicate spots with a CV greater than 20% were repeated.

the signal level from duplicate spots were compared and where coefficient of variation was greater than 20% the samples were assayed again.

3.17 Reviewer comments
6. Lines 376-377: 23 GST- and 2 MBP-tagged antigens were excluded from the microarray because of high signal from the corresponding protein tag control samples. Does this mean authors excluded 25 measurements or more generally they excluded 25 antigens from all multiplexed measurements as I understand they did? Authors should specify the criterion for exclusion in a quantitative way.

Author response

We observed high responses to the MBP and GST tags. It is likely that the high responses to MBP and GST were due to technical problems such as contaminating bacterial proteins since MBP and GST tagged antigens and the tags themselves were expressed in *E. coli*.

MFI Tag subtraction for GST or MBP tag resulted into negative values that were difficult to interpret. We thus excluded these proteins from the from the analysis.

Please see page 19 line 417 -422 We have included the following

GST and MBP tags had high MFI values such that post tag subtraction individual responses were negative, preventing accurate interpretation. This may have due to contaminating bacterial proteins since MBP and GST tagged antigens and the tags themselves were expressed in E. coli. Thus, data from 23 antigens tagged with GST and 2 with MBP were excluded from the analysis.

3.18 Reviewer comments
7. Line 388. I can understand that a "modified Poisson regression" was used to suit a binary outcome and be able to calculate relative risks from regression coefficients. Please explain this in the methods

Author response

Please see page 18 line 432

We revise the sentence as follows

A modified Poisson regression model (48) which has a robust error variance and provides relative risk as the parameter of primary interest and assist in convergence problems that sometimes arise with binomial regression models was used to determine the relative risk of treatment (due to clinical symptoms and or parasite densities >500 parasites/µl of blood) while adjusting for potential confounders, year of study, Dantu and α-Thalassemia genotypes, and anti-malarial drug levels.

3.19 Reviewer comments
8. Lines 391-394. Lasso is not a regression method, but a penalisation (a.k.a. regularisation) that is applied to a given regression. We do not know what type of regression authors are conducting here. Because the outcome is binary, one would expect they used a lasso-

regularised logistic or modified Poisson regression. They could also have conducted a lasso-regularised Cox regression. Or, more difficult to justify, a lasso-regularized linear regression. Authors should specify which type of lasso regression they used.

Author response

We regret this error, we used LASSO penalised regularization method, which is a penalised logistic regression. This has been corrected accordingly.

Please see line 428 page 19

Least Absolute Shrinkage and Selection Operator (LASSO)(48), which is a penalised regularization method, was used to determine the minimum number of antigens that account for the highest variance between two outcome groups.

3.20 Reviewer comments

9. Lines 141. It is incorrect to say that the method for choosing the lambda through cross-validation was done through selecting its minimum value as it is stated. It probably was obtained from minimising the value of a certain error score or loss function, e.g. Poisson deviance?

Author response

We have corrected this as below to show that the error that was minimised in this approach was the binomial deviance.

Please see page 7 line 141 we correct the sentence to read as

We first selected the optimal lambda through a cross-validation analysis that involved sampling with replacement of 15 variables at a time

3.21 Reviewer comments

10. Lines 149. "fitting decision trees for each sample, and noting the out-of-bag error for each data point " This description is a little confusing. Here, I presume authors mean that they fit a decision tree for each bag of samples (random subsets of the data sampled with replacement) and noted the out-of-bag error from its prediction performance on the left-out samples. Please clarify.

Author response

We regret that this was confusing. We have corrected this to read as below.

"*A random forest model was fitted to the data with bagging, i.e. repeatedly sampling from the dataset with replacement, fitting decision trees for each out-of-bag sample, and noting the out-of-bag error." Please see page 8 line 148*

3.22 Reviewer comments

11. Lines 401: The authors should specify what type of out-of-bag error measure was optimized in the hyperparameter tuning using cross-validation

Author response

We regret that this was not clear or specified. We optimised the misclassification rate also known as the out-of-Bag Error Rate.

Please see page 21, line 471

3.23 Reviewer comments
12. What is the distance metric used in the hierarchical clustering of Figure 2? If it is a Euclidian distance, then whether data was log transformed or not prior to clustering can make a considerable difference. It is odd that the unsupervised algorithm does not cluster according to CI and NI. Do the authors have any explanation?

Author response

We used VSN transformed data and Euclidian distance. Our data was initially VSN transformed prior to clustering to cater for systematic bias.

Malaria is a complex pathogen with over 5000 proteins. Given this complexity and the fact that we tested only a prioritised subset of 70 proteins, the fact that we saw >60% clustering is encouraging. The correlate of protection remains unknown and indeed immunity is only acquired slowly over repeated exposures. Here we only assess a subset of proteins among individuals with varied levels of exposure.

3.24 Reviewer comments
13. Figure 4C legend. There seems to be a preposition “of” missing

This has now been corrected

Reviewer #4 (Remarks to the Author):

I co-reviewed this manuscript with one of the reviewers who provided the listed reports. This is part of the Nature Communications initiative to facilitate training in peer review and to provide appropriate recognition for Early Career Researchers who co-review manuscripts.

REVIEWERS' COMMENTS

Reviewer #1 (Remarks to the Author):

I would like to thank the authors for the changes they have made which in my view have improved the manuscript. While it is unfortunate that the revised manuscript still lacks functional data that demonstrates that the identified candidates are causally linked to protection or that at least one of these can induce antibodies with some functional activity, I understand that experiments demonstrating such causality would require substantial time and resources. I appreciate that the authors have changed some of the wording to more accurately reflect the findings and tone down on identification of new vaccine candidates. Further to the proposed changes, I would like to suggest to also change the title accordingly, to put less emphasis on new vaccine candidates and more focus on the actual findings in the current work. I have no further comments.

Author response

We agree with this suggestion and have therefore revised the title of the manuscript to read as "***Controlled human malaria infection in adults identify combinations of merozoite antigens associated with clinical immunity***"

Reviewer #2 (Remarks to the Author):

All comments have been appropriately addressed, and the revised manuscript has significantly improved.

Author response

Thank you for the positive feedback

Reviewer #3 (Remarks to the Author):

I thank the authors for carefully considering my comments. The majority of my concerns were either addressed with additional plots or analyses or, at the very least, discussed and clarified in the manuscript.

However, given that the authors adopted an analytical approach based on assessing CI prediction –no modelling or causal inference framework-- I still don't understand why they didn't show ROC curves for CI classification, providing thus information on sensitivity and specificity of their classification, and, by this means, also reporting quite transparently how good their high vs. low antibody response threshold was, since ROC curves would have involved shifting this. Additionally, their ROC curve estimates could have been cross-validated by creating different folds of participants, training their predictive models on one set of folds and testing them on the remaining set recursively.

Author response

We agree that including a ROC analysis may have some additional value. We now include within the supplementary ROC curves for CI classification, indicating the sensitivity and specificity of our classification following the Poission regression analysis. This shows the strength of our high vs. low antibody response threshold classification.

Please see supplementary figure S4 where we include figure B. Please see supplementary figure below. We additionally repeat this for our sensitivity analysis and include this as a supplementary figure S7C.

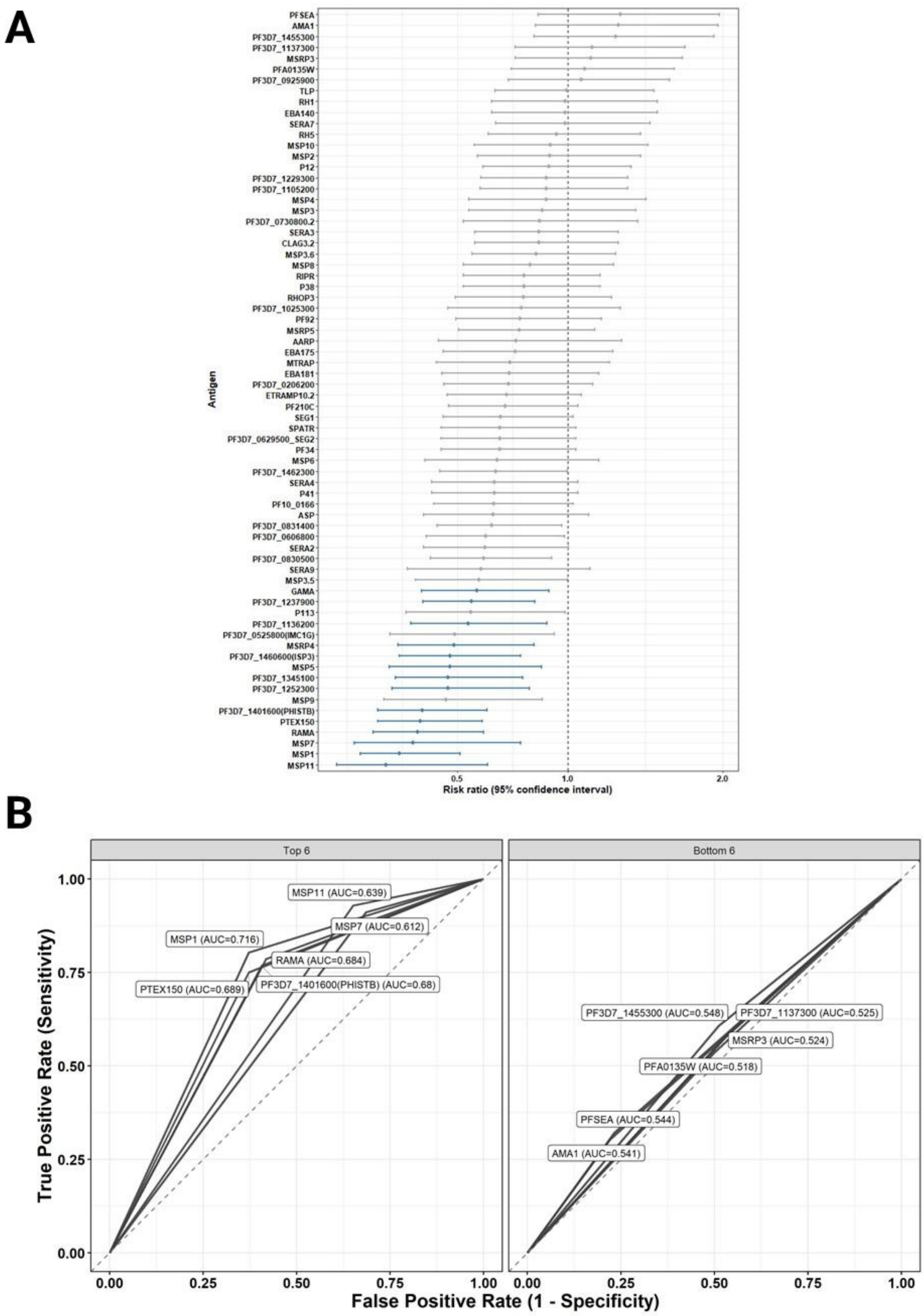

The authors mention their internal cross-validation of the Random Forest; however, this refers to the bootstrap sampling of features and observations (OOB error) used for hyperparameter tuning. This does not replace independent k-fold cross-validation at the participant level, which is necessary to generate unbiased estimates of predictive performance and to justify the 100% protection claim made in the abstract --100% which in immunology sounds inevitably shocking and unheard of, and still more for a pathogen such as malaria for which immunity is, to a great extent, acknowledged as partial.

I leave to the editor whether to push or not for this important methodological requirement. Apart from this, the manuscript has now considerably improved. I wish the authors the best of luck with the reception of their important work and its continuation.

Author response

We agree with this comment and have thus revised the manuscript to remove all claims of 100% protection. Please see abstract line 39. In addition, please see lines 181, and 191.

Further to this we explain within the discussion the fact that our observation of 100% protection requires further study and may not necessarily translate to vaccine efficacy. This study is within exposed adults and immunity significant varies in young children and naïve populations who are the target groups for malaria vaccination.

Please see page 12 line 254-257 we include *"We do not suggest that these combinations would likely translate to 100% protection in the field, the finding of complete protection in our setting relates to a controlled inoculum in 142 adults, and hence refers to our observed outcome which justifies the prioritization of these antigens. Vaccine efficacy would need to be measured in a trial."*

Reviewer #4 (Remarks to the Author):
I co-reviewed this manuscript with one of the reviewers who provided the listed reports. This is part of the Nature Communications initiative to facilitate training in peer review and to provide appropriate recognition for Early Career Researchers who co-review manuscripts.

Additional editorial edits

1. **Author affiliation**
   We add Modernising Medical Microbiology, University of Oxford, United Kingdom to the affiliation of Philip Bejon

2. **Abstract:** We extensively revise the abstract to meet editorial suggestions. It now reads as

"Two vaccines are currently licensed against Plasmodium falciparum malaria but offer only partial protection that wanes, necessitating repeated dosing that is challenging to implement where it is needed most. We previously identified a subset of Kenyan adults with naturally acquired clinical immunity using controlled human malaria infection studies (CHMI, n = 86/142; ClinicalTrials.gov - NCT02739763). Subsequent studies showed that clinical immunity was strongly correlated with IgG Fc-mediated functional activity against whole merozoites. Here, to zoom in on specific targets associated with clinical immunity, we use samples from the CHMI study and a custom protein microarray (KILchip) to measure IgG antibody reactivity against seventy recombinant merozoite antigens. We identify the top-ranking antigens by analyzing the data using five distinct statistical methodologies: the nonparametric Wilcoxon rank sum test, a Cox proportional hazards model, a modified Poisson regression model, and the machine learning Least Absolute Shrinkage and Selection Operator (LASSO) and random forest models. Antibodies against MSP1, MSP11, RAMA, MSP7, PF3D7_1401600 (PHISTB) and PTEX150 were consistently associated with clinical immunity and in combination predicted complete protection.

These findings may be useful to prioritize the next generation of malaria blood stage vaccine candidates."

3. Line 78: We delete "the" for clarity

4. Line 81: We delete "the" for sentence clarity

5. Line 99-102: we revise sentence to read as "*We previously explored four functions for normalization (variance stabilization normalization (VSN), Log_2, robust-linear-model (RLM) and cyclic loess) to identify one that optimally reduced the mean–variance dependence (MVD) (23)*

6. Line 129: We delete MSP2 this is a carryover from previous manuscript revisions. The sentence now reads "*Notable antigens identified in this first analysis included well-studied merozoite surface proteins 1 (MSP1), but not apical membrane antigen 1 (AMA1), MSP-3 or PfRh5*" instead of "*Notable antigens identified in this first analysis included well-studied merozoite surface proteins 1and 2 (MSP1 and MSP2), but not apical membrane antigen 1 (AMA1), MSP-3 or PfRh5*"

7. Line 181: We revise heading "Combination analyses predict complete protection" deleting 100%

8. Line 190: We revise 100% protection to complete protection

9. Line 212: We revise to *P. falciparum*

10. *Line 244:* We delete "*being the most*". The Sentence now reads as *Six merozoite antigens were consistently identified as strongly associated with protection using the five analytical methods: MSP1, MSP11, MSP7, RAMA, PTEX150 and PF3D7_1401600 (PHISTb).*

11. *Line 254. We include within the discussion "We do not suggest that these combinations would likely translate to 100% protection in the field, the finding of complete protection in our setting relates to a controlled inoculum in 142 adults, and hence refers to our observed outcome which justifies the prioritization of these antigens. Vaccine efficacy would need to be measured in a trial."*
12. *Line 262 of the discussion we revise the dN/ds ratios reported. The sentence now reads as "An analysis of dN/dS ratios against those of the highly conserved PfRh5 antigen (0.73) revealed that except for MSP1 (3.59) and MSP7 (2.9.)."* This is to harmonise to current ratios reported within the results following the revised analysis suggested by reviewer 2

13. *Line 291. We delete multiple antigens constructs are a challenge in vaccinology. And revise the sentence to "A vaccine strategy combining 6 antigens is likely not tractable given the challenges of multi-antigen formulations*."

14. *Line 335 we add "and"*
